# Conserved residue clusters at protein-protein interfaces and their use in binding site identification

**DOI:** 10.1186/1471-2105-11-286

**Published:** 2010-05-27

**Authors:** Mainak Guharoy, Pinak Chakrabarti

**Affiliations:** 1 Bioinformatics Centre, Bose Institute, P-1/12 CIT Scheme VIIM, Kolkata 700 054, India; 2 Department of Biochemistry, Bose Institute, P-1/12 CIT Scheme VIIM, Kolkata 700 054, India

## Abstract

**Background:**

Biological evolution conserves protein residues that are important for structure and function. Both protein stability and function often require a certain degree of structural co-operativity between spatially neighboring residues and it has previously been shown that conserved residues occur clustered together in protein tertiary structures, enzyme active sites and protein-DNA interfaces. Residues comprising protein interfaces are often more conserved compared to those occurring elsewhere on the protein surface. We investigate the extent to which conserved residues within protein-protein interfaces are clustered together in three-dimensions.

**Results:**

Out of 121 and 392 interfaces in homodimers and heterocomplexes, 96.7 and 86.7%, respectively, have the conserved positions clustered within the overall interface region. The significance of this clustering was established in comparison to what is seen for the subsets of the same size of randomly selected residues from the interface. Conserved residues occurring in larger interfaces could often be sub-divided into two or more distinct sub-clusters. These structural cluster(s) comprising conserved residues indicate functionally important regions within the protein-protein interface that can be targeted for further structural and energetic analysis by experimental scanning mutagenesis. Almost 60% of experimental hot spot residues (with ΔΔG > 2 kcal/mol) were localized to these conserved residue clusters. An analysis of the residue types that are enriched within these conserved subsets compared to the overall interface showed that hydrophobic and aromatic residues are favored, but charged residues (both positive and negative) are less common. The potential use of this method for discriminating binding sites (interfaces) versus random surface patches was explored by comparing the clustering of conserved residues within each of these regions - in about 50% cases the true interface is ranked among the top 10% of all surface patches.

**Conclusions:**

Protein-protein interaction sites are much larger than small molecule biding sites, but still conserved residues are not randomly distributed over the whole interface and are distinctly clustered. The clustered nature of evolutionarily conserved residues within interfaces as compared to those within other surface patches not involved in binding has important implications for the identification of protein-protein binding sites and would have applications in docking studies.

## Background

The analysis of sequence conservation in a protein family is a useful method for identifying residues that are functionally important - for catalytic activity or binding, or responsible for providing stability to the folded structure [1-10]. Residues comprising protein-protein interaction sites are very often found to be more conserved over those residing in the remaining surface [11-14]. Furthermore, within a given interface, core residues are usually conserved to a greater extent than the rim residues [15,16]. Binding surfaces on proteins are subjected to considerable selective pressure to maintain critical interactions with partner molecules throughout the course of evolution, and not surprisingly therefore, the use of residue conservation has been widely adopted in the identification of protein binding sites [17-20]. In addition to the conservation of individual interface residues, conservation of interacting residue pairs have also been found to characterize protein-protein binding sites [21].

The question addressed in this paper is whether the subset of conserved residues in a protein-protein interface occurs scattered across the interface, or cluster together in three-dimension? It is possible that the conserved residues would form one or more localized clusters within the interface as it would enable the formation of "functional motifs". It has recently been shown in protein-DNA interfaces that the most stabilizing residues (putative 'hotspots') are those that form clusters of conserved residues at the interface [22]. The residues in these clusters are more tightly packed than those in the remainder of the interface and analysis of experimental mutational data suggests the existence of cooperative interactions between them (which makes these clusters of conserved residues contribute significantly more towards the stability of the interaction as compared to isolated conserved residues). Such correlation between clustering of conserved residues and functional importance of that region is often found to be a recurring theme in the study of protein structures. For example, spatial clustering of conserved residues yields information about the observed functional site in individual proteins and also enables large-scale functional annotation by transfer of function from a characterised protein to a homologue of unknown activity [23]. Such clustering improved predictions in the case of enzyme active sites [24]. Clusters of evolutionary conserved residues are also commonly observed within protein tertiary structures serving both structural and functional roles [7,25,26]. How common is this for protein interfaces? A thorough analysis of this phenomenon in different types of protein-protein interfaces would be of use in the prediction of binding sites. These conserved residue clusters may be analogous to modules containing conserved and highly cooperative groups of interface residues that characterize binding sites [27,28].

Of the large number of residues comprising a protein-protein interface, only a few contribute significantly to the free energy of binding. These "hot spot" residues are generally occluded from bulk solvent, being surrounded by other less important residues [29]. It is probable that a significant fraction of these experimentally determined hot residues would be localized within the conserved residue clusters. Therefore, the identification of these clusters would be a useful guide for mutational studies to pinpoint the appropriate "functional determinant" regions. Indeed, computational hot spot residues, instead of being uniformly distributed across the interface, occur as clusters of tightly packed regions [30]. In this work we show that the conserved residues are significantly clustered in the interface and this fact can be used as a search tool to identify the possible binding patch in the structure.

## Methods

### Datasets of protein-protein interfaces

The sets of interfaces used were 122 homodimers [31] and 204 heterocomplexes [32] - the former set contains obligate dimers with two identical chains and the latter group comprises individually stable proteins that bind to their partner proteins and may again separate depending upon the physiological conditions existing within the cellular environment. For each PDB [33] file containing the structural coordinates of the protein complex, a list of interface residues was generated using ProFace [34]. Atoms/residues from both partners that lose more than 0.1 Å^2 ^of surface area upon complexation are considered as belonging to the interface [35].

### Measuring sequence conservation

The sequence variablility at each interface residue position is calculated as the Shannon entropy (s) in sets of homologous protein sequences [15]:(1)

where, p_i_(k) is the probability that the i^th ^position in the multiple sequence alignment is occupied by a residue of class 'k', and s(i) is the sequence entropy of that position. A low value of sequence entropy, s(i) implies that the position has been subjected to relatively higher evolutionary pressure than another position in the same alignment having a higher sequence entropy value. Multiple sequence alignments were obtained from the Homology-Derived Secondary Structure of Proteins (HSSP) database [36]. The database provides for each PDB file an alignment of protein sequences deemed structurally homologous to the query protein on the basis of a homology-threshold curve. While using Eq. 1 the amino acids were grouped into 7 classes based on the similarity of the environment of each amino acid residue in protein structures, and mutations within a given class were assumed to be conservative and did not attract a penalty [15]. The amino acid groups were as follows: (1) Ala, Val, Leu, Ile, Met, Cys; (2) Gly, Ser, Thr; (3) Asp, Glu; (4) Asn, Gln; (5) Arg, Lys; (6) Pro, Phe, Tyr, Trp; and, (7) His.

Eq. 1 makes use of the probability (or frequency) of occurrence of each residue class in a given aligned position. However, it does not take into account the "background" frequencies of these amino acids. It has been shown previously that the use of background frequency information significantly improves entropy-based functional site prediction within protein structures [37]. In order to evaluate whether such a scheme improves the results in the present study, we modified Eq. 1 as follows:(1a)

where p_back_(k) denotes the background frequencies of the amino acids in group 'k', and the remaining terms are the same as in Eq. 1. This relative entropy measure (also called Kullback-Leibler divergence) is similar to the one used by Wang and Samudrala [37]. A higher deviation from the "background" indicates a stronger level of constraint in evolution, indicating a possibly important functional role for that position. Since we partition the residue space into 7 groups, we calculated the background frequencies for each of these groups and incorporated them into Eq. 1a. Only the residue types that occurred in a given aligned column were used in computing the relative entropy for that position. We also had to decide whether to calculate the background frequencies using the overall protein sequence or use a particular subset (such as the interface region). Choosing an appropriate "background" was important because the sequence composition of interfaces differs from that of the overall protein. We want to identify conserved residues in the interface and a background calculated from overall protein sequences may result in incorrect assignments. On the other hand, a background calculated from the sequence composition of interface residues will correctly increase the conservation signal for invariant positions containing residues that are "rare" for interfaces, but which may not be "rare" in overall protein sequences. Therefore, we calculated the background frequencies using interface residues belonging to complexes of the Docking Benchmark 3.0 [38]. We also compared using frequencies from the overall protein sequences, but better results were obtained using interface sequences alone.

### Identification of conserved interface residues

For each interface with 'n' residues an average value of sequence entropy was calculated:(2)

We used three different criteria with increasing levels of stringency to identify the conserved interface residues, and compared the results. (1) Interface residues with sequence entropy values lower than the average (< s>_int_) were assumed to constitute the conserved residues. (2) We also selected the subset of conserved residues with sequence entropy lower than the average less the standard deviation (< s>_int _- σ). It may be mentioned that the values of the mean and standard deviation used for selecting the set of conserved interface residues were calculated for each individual interface. (3) Finally, we also used only those residues with the sequence entropy value of 0.0, i.e., the fully conserved residues.

### Measure of the degree of spatial clustering (M_s_)

The degree of spatial clustering of a set of residues can be measured as the average inverse distance between all pairs of positions in that set [25]:(3)

where N_s _is the number of residues in the set, N_pairs _is the number of different pairs of residues in the set given by: N_pairs _= (N_s_-1).N_s_/2; and, r_ij _is the distance between the centers-of-mass of the two residues in question, i and j. Greater the value of M_s_, greater is the degree of spatial clustering of the residues in the set. The advantage of this inverse-distance based formula is that one or a few outlier positions are unable to significantly influence the overall value of M_s _for the entire set. The values of M_s _that are obtained are continuous and can be used in ranking different sets of residues.

For each interface Eq. 3 was employed twice, once for the subset of conserved residues (M_s,cons_) and then for the whole interface (M_s,int_). The contrast between the spread of inter-residue distances between the two sets (conserved residues versus all interface residues), ρ, is an indicator of the extent of clustering of evolutionary conserved residues,(4)

ρ > 1.0 indicates that the subset of evolutionary conserved residues is clustered within the interface. This gives us a single numeric value representing overall whether or not (and to what extent) the conserved residues are clustered within the interface (Eq. 3 down-plays the effect of one or few outlying isolated conserved residues and gives a more general idea of whether the conserved residues are grouped together or scattered in the interface region). However, the occurrence of isolated conserved residues has been dealt with while considering cluster size.

### Assessment of significance of clustering of evolutionary conserved residues by comparison to random subsets of residues

The degree of clustering of conserved interface residues (M_s,cons_) was compared to M_s _values obtained for 1000 random subsets of interface residues of the same size in each structure. The average (and SD) of the M_s _values calculated for the 1000 random subsets (denoted by < M_s,random_>) was compared to M_s,cons _obtained for each interface.

### Identification of sub-clusters of conserved residues

Compared to the overall interface, the conserved residues were found to be spatially clustered; but within this set, spatially distinct sub-groups of conserved residues that formed sub-clusters could often be discerned (including single isolated conserved residues or conserved 'singlets' as described by Ahmad et al. [22]). To identify if one or more such sub-clusters are formed, the average linkage method used earlier to identify interface patches [35] was used. The algorithm involves the setting of a threshold distance. Threshold distances of 21 and 15 Å for homodimers and complexes respectively, were selected for identifying the number of sub-clusters. These cutoffs correspond to half the average value of the maximum distance between any two atoms belonging to conserved residues in all the interfaces. All interfaces were then visually checked for the occurrence of the sub-clusters.

### Experimental alanine scanning data and conserved residue clusters

The clustering analysis was also carried out on a set of 26 protein-protein complexes for which experimental alanine scanning mutagenesis on the interface residues has been carried out. The list of complexes used has been described in our earlier paper [39]. Interface residues with experimental ΔΔG values of ≥ 1, ≥ 1.5, and, ≥ 2 kcal/mol were collected and the fraction of these residues that occurred within the conserved clusters were found out.

### Generation of surface patches and evaluation of the clustering of conserved residue positions in the interface vis-à-vis surface patches

Three different procedures were used for the identification of surface patches. Method 1: NACCESS [40] was run on the atomic coordinates of the protein subunit (or chain) and residues with relative surface accessibility ≥ 5% were selected as residing on the protein surface. Each surface residue (represented by its center of mass) was taken in turn and all the other surface residues within a fixed radius were selected as belonging to the surface patch with the original residue as the center. The average maximum distance between two atoms of a standard size interface is 30 Å for complexes [35] and 44 Å for homodimers [31]. Accordingly, half of the above values, 15 and 22 Å, respectively, were the radii used to generate surface patches for complexes and homodimers. The procedure thus defined a number of contiguous, overlapping patches of surface residues, roughly similar in size to the interface region. Conserved residues within each patch were then selected and the M_s _values (Eq. 3) for both the conserved and the overall residues in the patch were computed. The procedure was repeated for each patch. Finally, the surface patches were arranged in descending order of ρ (Eq. 4) and the rank of the true interface in relation to all the other surface patches was found out.

Two variations were also explored in the algorithm used to generate surface patches. Method 2: Instead of using standard cutoffs for all the proteins in the dataset, individual cutoffs were used for each protein depending on the size of the particular interface. For each interface the maximum distance between any two atoms was found out and the radial cutoff was set as half that value. This step is likely to generate surface patches of a size which will more closely approximate the size of the true interface, than a cutoff based on the average value calculated over the whole database. Method 3: In addition to using individual cutoffs for each protein, vector constraints were used while selecting surface neighbors around each central residue [41]. This step avoids generating surface patches that include residues from "opposite sides" of a protein molecule. In this step, a 'solvent' vector (pointing into the solvent) is calculated for each surface residue of the protein. A particular surface residue is taken and the centre of gravity of its nearest ten residue neighbors is calculated. The vector from the center of mass of the particular surface residue to this center of gravity was then calculated - the inverse of this (pointing into the solvent) is called the 'solvent' vector. Each surface residue was assigned such a vector. When generating the surface patch, a particular residue is included in the patch if the angle between the solvent vectors of the residue and the central residue was < 110°.

All three definitions of the surface patches result in approximately contiguous, circular regions of the protein surface which overlap each other. We also evaluated how the generated patches sampled the true interface region by calculating a percentage overlap - fraction of residues common between the real interface and the surface patch relative to the total number in the interface:(5)

where NrI is the number of residues in the true interface patch, and NrC is the number of residues in the generated surface patch. The numerator defines the set of residue common between the real interface and the calculated patch.

## Results

### Clustering of conserved residue positions in protein-protein interfaces

**Figure 1:**
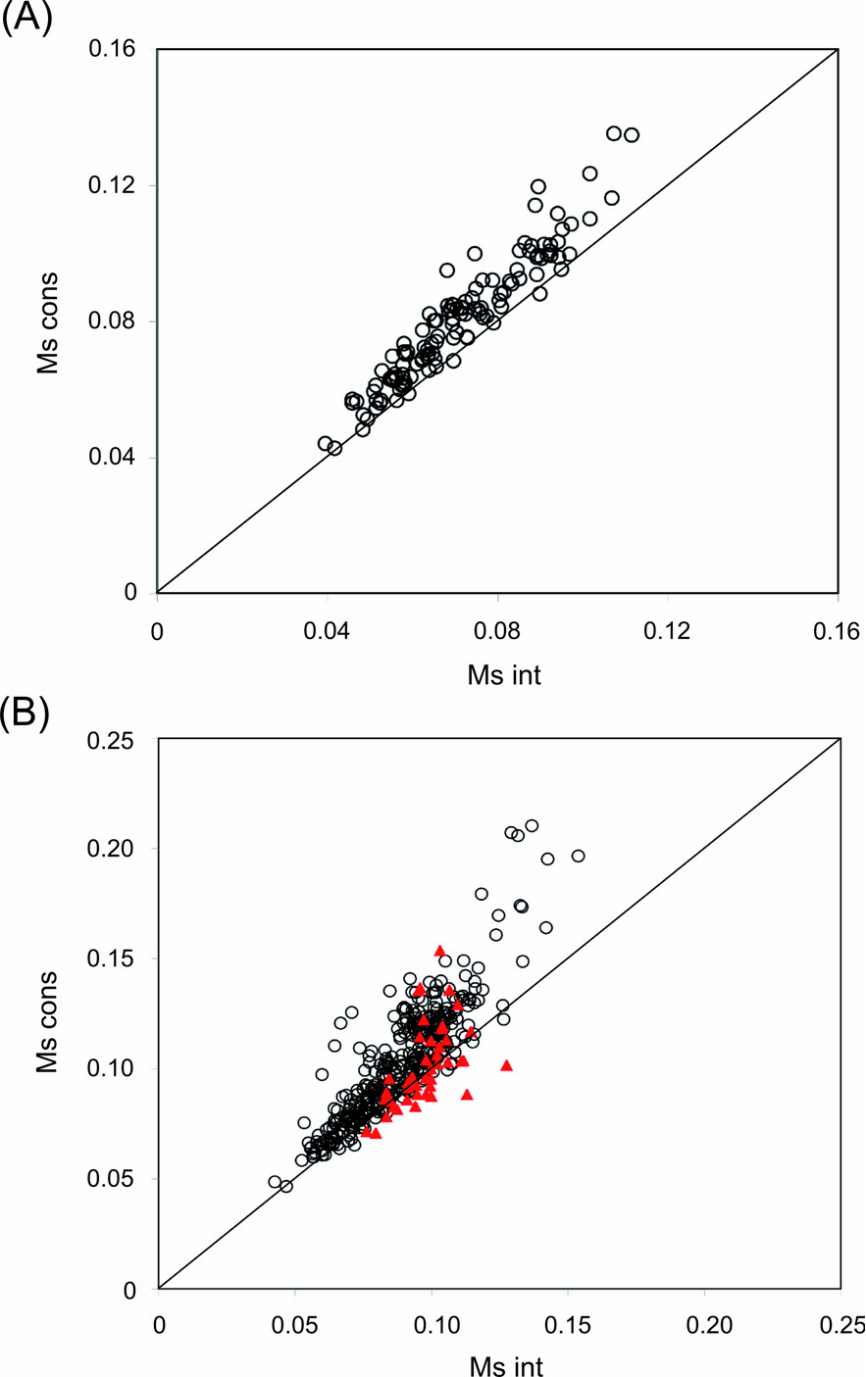
**Distribution of M_s _values for the conserved subset of interface residues (M_s,cons_) versus that for the entire set of interface residues (M_s,int_) for (A) homodimers and (B) protein-protein complexes**. In (B) the antibody-antigen complexes are marked as triangles.

The first issue addressed is the relative spatial location of evolutionary conserved residues in protein-protein interfaces, if these are scattered throughout the interface or they form spatial clusters. M_s _(Eq. 3) is a simple but useful measure for assessing the degree of spatial clustering of a group of points (residues in this case) in space [25]. Since M_s _uses an inverse distance relationship, residues that are close together will mainly influence its value and one or a few outliers will not unduly affect it. A high value of M_s _indicates that the set of residues under consideration are mostly clustered together. M_s _is calculated both for the subset of conserved residues as well as for the whole set of interface residues. The ratio (ρ) of M_s _for the conserved subset to that for the entire interface gives an indication of the clustered (or dispersed) nature of the distribution of the evolutionary conserved subset. ρ > 1.0 indicates that the conserved residues are relatively more clustered compared to the whole interface. A few representative examples of interfaces where the evolutionary conserved residues are clearly clustered together are shown in Additional file 1, Figure S1. Overall, the same picture holds true, as can be seen in Figure 1, where M_s,cons _and M_s,int _for each interface are plotted. A point lying above the diagonal indicates that M_s,cons _is greater than M_s,int _(i.e., ρ > 1.0), implying that the conserved residue subset is more clustered in space relative to the overall interface (the detailed values are provided in Additional file 1, Table S1). For 96.7% (117/121) and 86.7% (340/392) interfaces in homodimers and heterodimers, respectively, a ρ value of greater than 1.0 is obtained (Table 1). The overall difference between M_s,cons _and M_s,int _for both datasets is statistically significant at the 1% level indicating that the phenomenon is non-random and almost universal (P < 0.01 implies that the observed difference between M_s,cons _and M_s,int _is significant). We repeated the clustering analysis but this time the calculation of sequence entropies was carried out using Eq. 1a (which takes amino acid background frequencies into account). However, this additional step did not affect the clustering results significantly (values shown in square brackets in Table 1). Therefore, we restricted further analysis using Eq. 1 only.

**Table 1:** Parameters delineating the clustering of conserved residues in interfaces

Interface type	Average^a^			Number of interfaces^b^		
	
	M_s cons_	M_s int_	ρ	Total	With M_s cons _greater than M_s int_	*P *values^c^
Homodimers	0.081 (0.02) [0.079 (0.02)]	0.071 (0.02) [0.071 (0.02)]	1.13 (0.08) [1.11 (0.09)]	121	117 [108]	1.57E-04 [1.50E-03]
	
	0.087 (0.02)	0.070 (0.02)	1.24 (0.20)	103	94	1.93E-08

Complexes	0.102 (0.03) [0.101 (0.02)]	0.089 (0.02) [0.089 (0.02)]	1.14 (0.14) [1.13 (0.17)]	392	340 [308]	9.64E-14 [3.74E-11]
	
	0.113 (0.04)	0.090 (0.02)	1.26 (0.30)	309	252	< 2.2E-16

Complexes (antibody-antigen excluded)	0.103 (0.03)	0.088 (0.02)	1.16 (0.14)	348	313	4.86E-14
	
	0.115 (0.04)	0.089 (0.02)	1.28 (0.30)	271	229	< 2.2E-16

Antibody-antigen complexes	0.101 (0.02)	0.097 (0.01)	1.04 (0.15)	44	23	0.59
	
	0.103 (0.03)	0.097 (0.01)	1.07 (0.29)	38	21	0.57

Two sets of values are provided, corresponding to two different ways of identifying the subset of conserved residues (see Methods). In the first, conserved residues in each interface are those whose sequence entropy values (calculated using Eq. 1) are lower than the mean sequence entropy (< s>_int_) for that interface; in the second method, conserved residues have s < (< s>_int _- σ), σ being the standard deviation of 's' values over all residues in that particular interface. The first method was also repeated by using Eq. 1a (instead of Eq. 1) for the calculation of sequence entropy and the results are provided in square brackets.

a Standard deviations are in parentheses.

b Multiple sequence alignments were available in the HSSP database for all proteins in our datasets with the exception of one homodimer, and therefore the analysis could not be carried out for that interface. 121 homodimeric interfaces and 408 interfaces belonging to 204 protein-protein complexes were analyzed - since the subunit interfaces in homodimers are identical, the analysis was performed for only a single subunit in homodimers. For protein complexes, each of the two components was analyzed separately. The average numbers of aligned homologous sequences in the HSSP files were 768 and 1391 for homodimers and protein complexes, respectively, and the percentage sequence identities of the aligned proteins ranged between 30 and 100%. For 16 protein chains belonging to the dataset of complexes, all the interface residue positions in the multiple sequence alignments were fully conserved and therefore the average interface entropy was 0.0. This did not allow the identification of the subset of conserved residues within the whole set of interface residues, precluding the calculation of clustering of conserved residues relative to the whole interface. Therefore, the statistics are shown for the remaining 392 interfaces only. A smaller number of interfaces is reported in the second row of data (corresponding to Method 2), where because of the use of a more stringent condition of conservation, some interfaces, with 0 or 1 conserved residue, get excluded from consideration.

c The non-parametric Mann-Whitney U-test was used to test for statistical significance of the hypothesis that M_s,cons _is greater than M_s,int_. P < 0.01 indicates that M_s,cons _is significantly greater than M_s,int _at the 1% level. All statistical calculations (including P-values) were implemented using R [64].

We also carried out the same calculations using a more stringent criterion for selecting conserved interface residues (those with individual sequence entropy values [from Eq. 1] less than the average sequence entropy at the 1σ level). A fewer number of residues from each interface are labeled conserved, but the conclusion that the conserved residues are clustered within the interface remains the same (Table 1 and Additional file 1, Figure S2A,B). 91.3% (94/103) homodimers and 81.6% (252/309) complexes retain the characteristic tendency for conserved residues within protein interfaces to be clustered. The extent of clustering within the interface (given by ρ) actually increases when the stringency for selecting conserved residues is increased. Finally, to further test the robustness of the approach, we used the most stringent criterion possible for identifying conserved residues (those having sequence entropy equal to 0). The features of the distribution of data points remain the same (Additional file 1, Figure S2C,D). As such, in the subsequent sections we restrict ourselves to the results obtained using the first method.

We had previously shown that antibody-antigen complexes are not good candidates for analysis based on evolutionary conservation because of high rates of mutation at the interface regions necessary for antibodies to recognize a wide arsenal of antigens [15]. This is also reflected in the present analysis. Figure 1B shows that a large fraction of antibody-antigen complexes are located either below or on the diagonal line, showing that the clustering of 'conserved' interface residues in these complexes is less clear compared to the general dataset. Table 1 also shows that there is an improvement in the statistics when antibody-antigen complexes are separated out from the general dataset of complexes.

**Figure 2:**
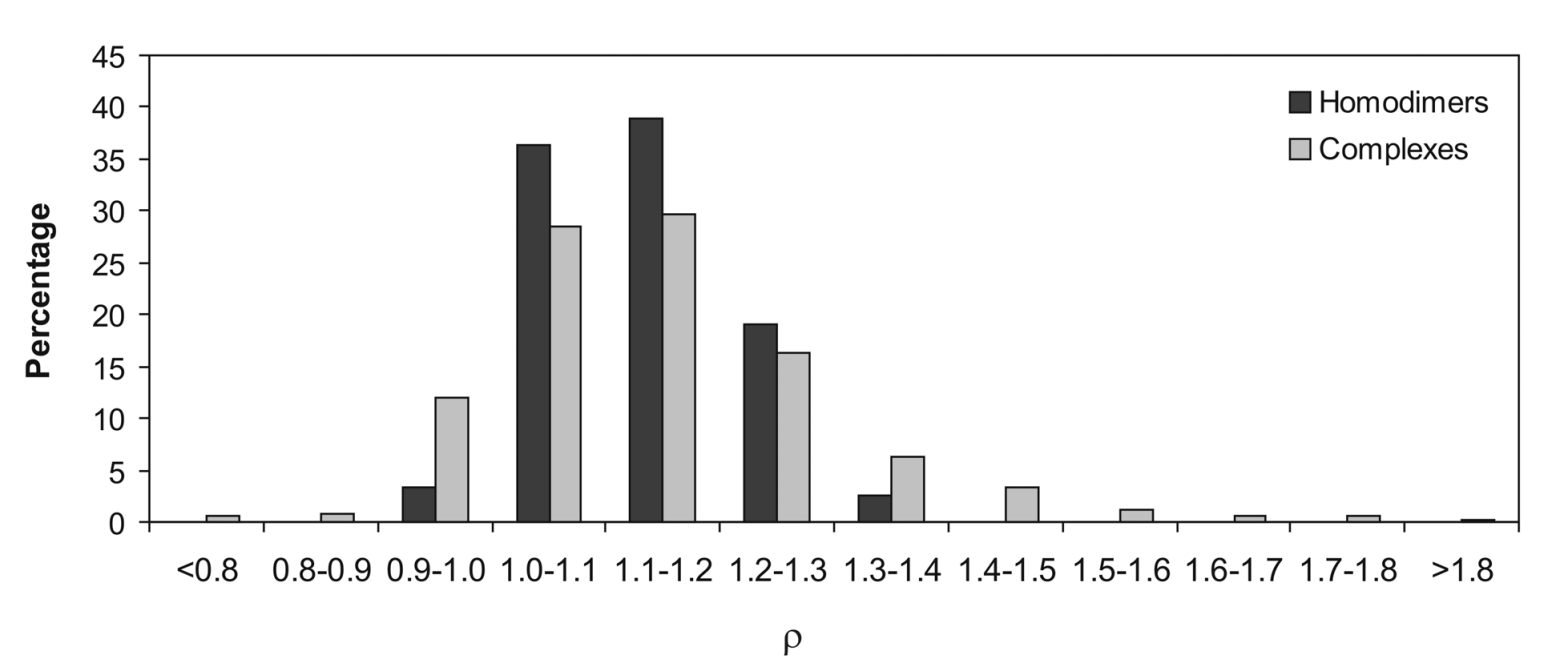
**Histogram showing the percentage distribution of the ρ values for the interfaces in homodimers and protein-protein complexes**.

Figure 2 shows the distribution of the ρ values for the interfaces in homodimers and protein complexes. Almost three-quarters of the homodimeric interfaces have ρ values between 1.0 and 1.2. In case of the complexes, more than 50% of the interfaces belong to this range. The range of ρ values extends up to a much higher range in case of complexes compared to homodimers. Two examples of interfaces belonging to the protein complexes and having high ρ values are shown in Additional file 1, Figure S1B,C. Overall, this histogram shows that although individual interfaces show differences in their shapes and sizes and the absolute values of M_s,cons _and M_s,int _may vary within a certain range, for the majority of interfaces ρ > 1.0, implying that the group of conserved residues occurs clustered together rather than being scattered throughout the entire interface.

We also found that subsets of evolutionary conserved interface residues are significantly more clustered than what is observed in subsets of the same size consisting of randomly selected interface residues (Table 2 and Additional file 1, Figure S3). M_s _values were calculated for conserved residues within each interface (M_s,cons_). From the same interface, subsets of residues (of the same size as the number of conserved residues) were selected randomly and their M_s _values were found out. The average M_s,random _value of 1000 such random subsets were computed for each interface and compared against M_s,cons_. In 96.7% (117/121) homodimeric and 87.7% (341/389) protein complex interfaces, we found that the randomly selected groups of residues were indeed less clustered than the conserved residue subset in the interface, and this difference was statistically significant at the 1% level (*P *< 0.01).

**Table 2:** Statistics showing the significance of clustering of conserved interface residues compared to the clustering in the subsets of the same-size containing randomly selected interface residues from the same structure

Datasets	Selection of conserved interface residues^a^							
	
	s < < s>_int_				s < (< s>_int _- σ)			
	Number	M_s,cons_	< M_s,random_>	*P*-value^b^	Number	M_s,cons_	< M_s,random_>	*P*-value^b^
Homodimers	121	0.081 (0.02)	0.071 (0.02)	1.6E-04	103	0.087 (0.02)	0.070 (0.02)	2.0E-08

Complexes	389	0.103 (0.03)	0.089 (0.02)	6.5E-14	309	0.113 (0.04)	0.090 (0.02)	2.2E-16

a Conserved interface residues were selected using different criteria (see Methods and Table 1 footnote).

b *P*-values refer to the significance levels for the Mann-Whitney U-test corresponding to the hypothesis that M_s,cons _is greater than < M_s,random _> (i.e., the degree of clustering of conserved residues within an interface is greater than that of random, same-size subsets collected from the same interface). A *P*-value < 0.01 indicates that the difference is significant at the 1% level. Calculated using R [64].

### Size of the conserved subsets and variation with interface area

On average, the homodimer interfaces contain 27 (± 16) conserved interface residues per subunit (comprising 52 ± 29 interface residues), with the average interface area being 1941.2 (± 1108.2) Å^2^. For the protein-protein complexes, these numbers are: 15 ± 8 conserved residues (and 29 ± 13 interface residues) per chain, which on average possesses an interface area of 1000 ± 422 Å^2^. Since however, on average, homodimer interfaces are almost twice the size of protein complex interfaces [31], the numbers of conserved interface residues in each subunit (or chain) when normalized per 1000 Å^2 ^of the interface area were 13.9 and 14.9 for homodimers and complexes, respectively. The number of conserved interface residues (and the total number of interface residues) per subunit (or chain) as a function of the interface size has been plotted in Additional file 1, Figure S4. Both the number of conserved interface residues and the total number of interface residues correlate very well with interface size in case of homodimers, but the correlation is slightly inferior in case of protein complexes. Broadly, the number of conserved residues is about half the number of interface residues (as can be expected from the primary definition of conserved residues used in the study that selects positions with sequence entropy smaller than the interface average), making the slopes of the two plots different, but having very similar correlation of interface size with either the total number of interface residuess or the number of conserved residues.

### Formation of multiple conserved residue clusters in larger interfaces

**Figure 3:**
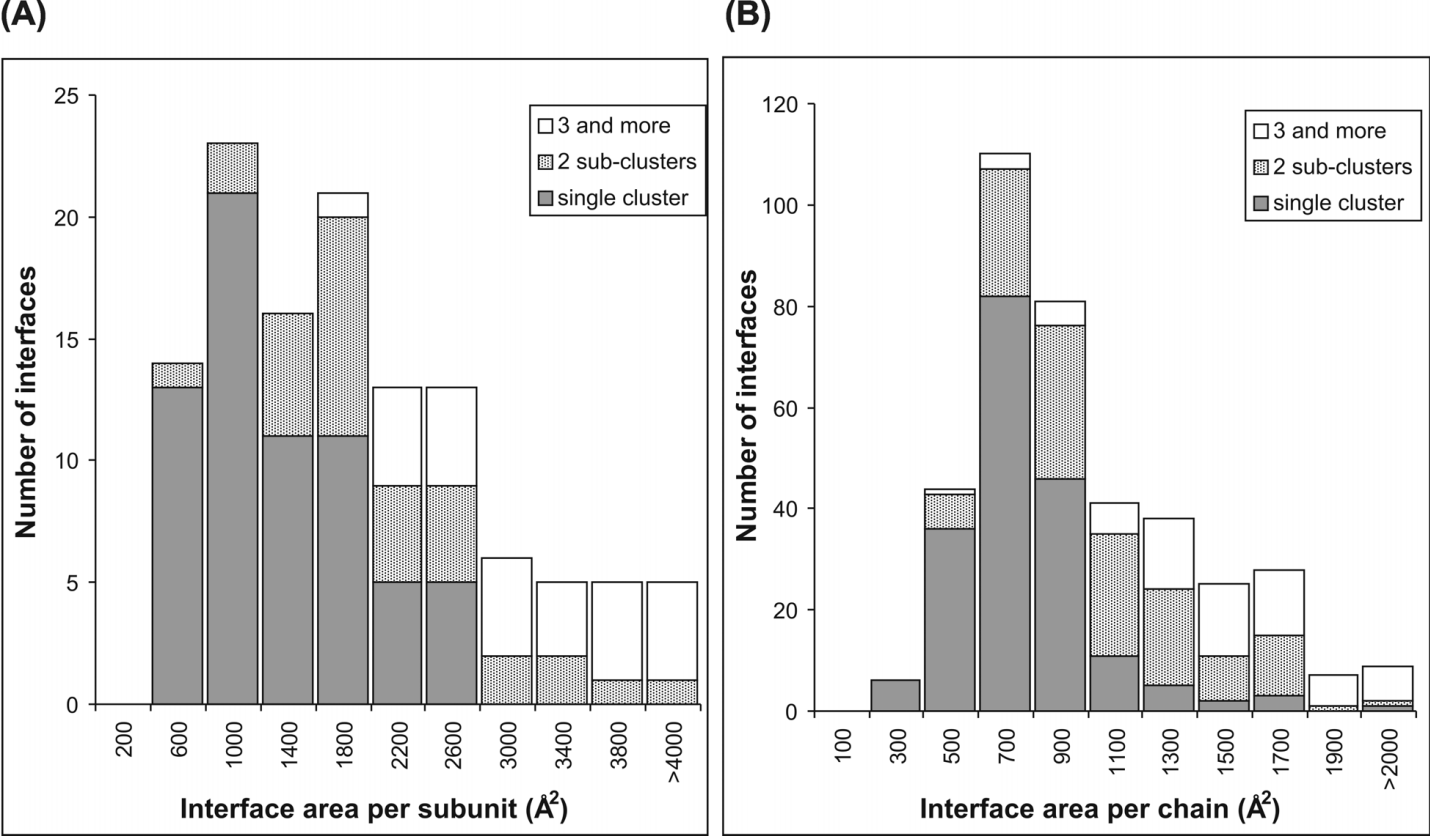
**Histogram of the distribution of the number of conserved residue sub-clusters in interfaces as a function of the interface size in (A) homodimers and (B) protein-protein complexes**. The x-axis labels mark the middle of the range in each column. Bins are of size 400 Å^2 ^in (A) and 200 Å^2 ^in (B). In (A) only the interfaces of subunit A have been considered because of the identical nature of the two chains. In (B), each component of the protein complex has been considered separately.

The larger interfaces often comprise of multiple distinct clusters of evolutionary conserved residues. The number of sub-clusters formed by the subset of conserved residues can be easily identified by a simple geometric clustering algorithm which uses the average linkage method [35] (see Methods). The clustering algorithm was run separately on the two polypeptide chains of the complexes, but on a single subunit only of the homodimers. Of the 121 interfaces in homodimers, 66 formed a single cluster of conserved residues; 31 had two sub-clusters and 24 possessed 3 or more. Similarly, out of 392 interfaces from the set of protein-protein complexes, 193, 130 and 69 respectively had 1, 2 or (3 and more) conserved residue sub-clusters (Figure 3; complete details given in Additional file 1, Table S1). Figure 3 also shows that the number of sub-clusters correlates well with the size of the interface. For the homodimers, all the single-cluster interfaces have interface areas < 2800 Å^2 ^and all but one of the interfaces with 3 or more sub-clusters possess interfaces larger than 2000 Å^2^. A similar observation can also be drawn from the complexes - out of the 193 single-cluster interfaces, 94.3% (182 cases) have interface areas within 1200 Å^2 ^whereas 54 of the 69 (~ 80%) interfaces with 3 or more sub-clusters have interfaces > 1200 Å^2^.

Examples of a few interfaces containing multiple clusters of conserved residues are shown in Additional file 1, Figure S5. These multiple structural sub-clusters containing evolutionary conserved interface residues may be contributed either by a single protein domain or from separate structural domains. For instance, in the first example of the cell signaling complex between human Rac and RhoGDI, although the interface of the latter protein contains two well-clustered sub-groups of conserved residues (Additional file 1, Figure S5A), the protein itself is composed of only a single domain (having the immunoglobulin-like β-sandwich fold with SCOP [42] classification b.1.18.8). In contrast, in the second example shown in Additional file 1, Figure S5B, the interface formed by the protein internalin-A contains two well separated conserved residue sub-clusters and each of them is contributed by a different structural domain. Internalin-A contains two domains - the first one containing an immunoglobulin-like β-sandwich fold and the other with a right-handed β-α superhelix leucine-rich repeat fold (with SCOP identifiers b.1.18.15 and c.10.2.1). Both domains of internalin (residues 36-416 and 417-496, respectively) participate in interface formation when it binds to its receptor E-cadherin. The cluster depicted in orange comes from the N-terminal domain, whereas the yellow-colored conserved cluster is formed from residues coming from both domains.

The next two examples depict homodimeric molecules. Once again, the multiple conserved sub-clusters may be part of the same protein domain or may come from distinct structural domains. The subunit interface of the enzyme glucosamine 6-phosphate synthase contains 3 distinct conserved clusters (2 larger ones and a smaller one) (Additional file 1, Figure S5C) and the protein itself is also composed of multiple domains of the α/β type. In another example, for the other interface shown in Additional file 1, Figure S5D, the protein contains four separate domains (an N-terminal domain with SCOP classification b.1.18.9, two identical C-terminal domains b.1.5.1 and a catalytic domain d.3.1.4). The N-terminal domain extends from residue numbers 5-190 and two sub-clusters (in red and blue) are contributed by this domain. The central catalytic domain (residues 191-515) also forms part of the interface and the third sub-cluster (yellow) is part of this domain. Finally, the fourth conserved residue sub-cluster (orange) is contributed jointly by the catalytic domain and the first of the C-terminal domains (residues 516-627).

### Cluster size

We also analyzed the distribution of the cluster size (Additional file 1, Figure S6). Conserved residues can occur singly, or form clusters (comprising of varying numbers of residues) with other conserved residues. Considering the datasets of homodimers and complexes, there are a total of 213 and 673 distinct clusters of conserved residues, respectively in the two types of interfaces. On average a cluster consists of 15 and 8 conserved residues in homodimers and complexes, respectively. Their distribution in terms of the cluster size (i.e., the number of conserved residues comprising each cluster) shows that there are only 6% (13/213) and 7.4% (50/673) of single isolated conserved residues. Therefore, it is clear that the majority of conserved residues prefer to be clustered together with other conserved residues rather than remain isolated.

### Preferred amino acid types in conserved residue clusters

**Figure 4:**
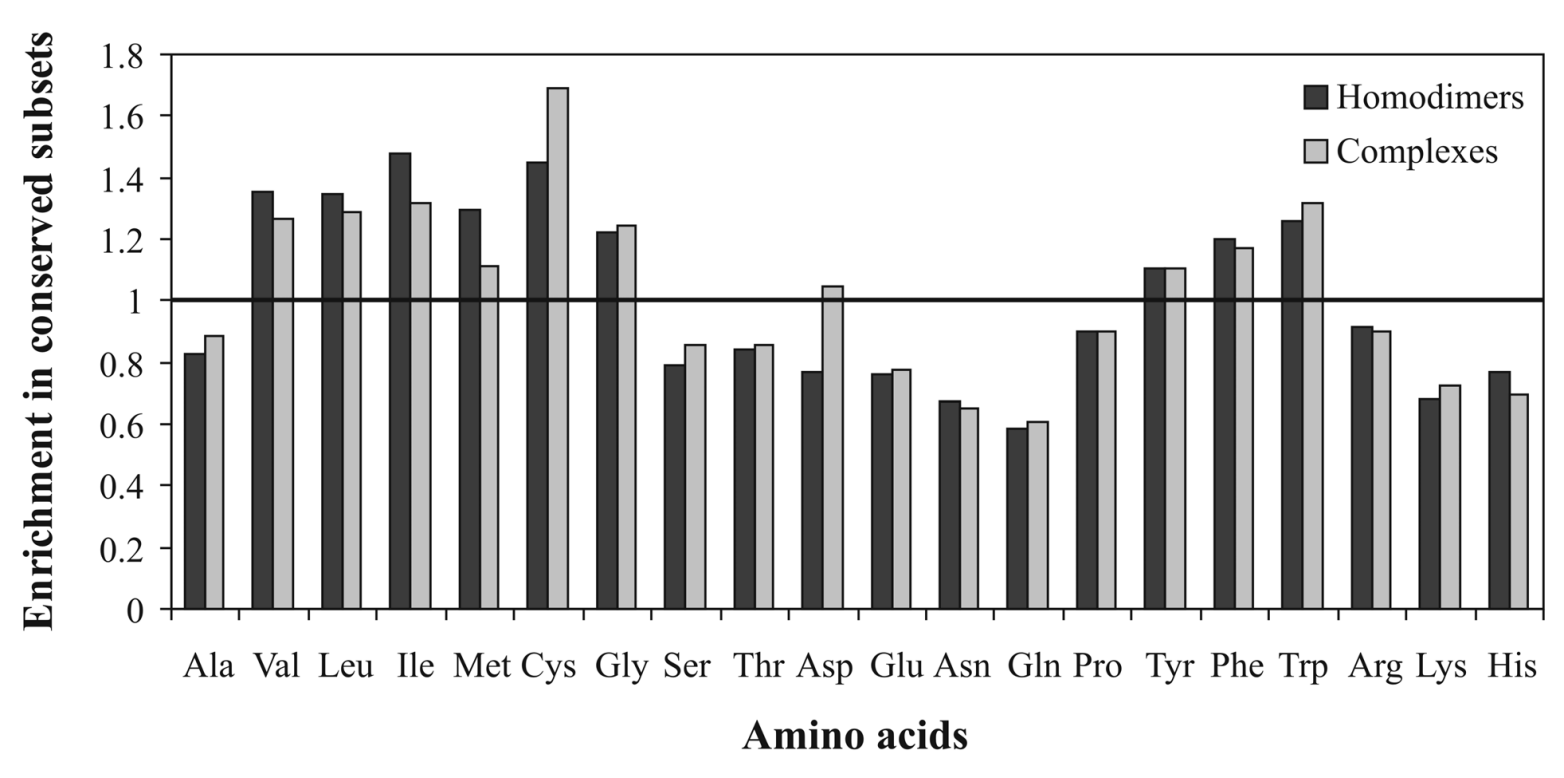
**Relative enrichment of the 20 amino acid types within conserved regions in protein-protein interfaces**.

Certain amino acid types are enriched in the conserved residue clusters. The relative enrichment of each of the 20 amino acid types in the conserved subsets compared to the overall interface has been calculated (Figure 4). The enrichment (E_X_) of a particular amino acid (X) is defined as the ratio of the frequency of occurrence of that amino acid in the conserved residue subset compared to its frequency in the whole of the interface region, i.e.,

The same types of residues are found to be preferred in conserved residue clusters in both homodimeric and protein complex interfaces, namely, hydrophobic (Val, Leu, Ile, Met), Cys, Gly, and the aromatic residues (Tyr, Phe, and Trp). Except for Gly the observed preference matches with the propensities of residues to occur in interface core [43]. The only distinction between the two datasets comes from Asp - this residue is disfavored in conserved clusters in homodimers, but slightly favored in complexes. It may be mentioned in this connection that of the two negatively charged residues, Asp is found more as binding hot spots in complexes [39]. Interestingly, Ala is the only hydrophobic residue that is under-represented in the conserved subset of interface residues. Charged (both positive and negative) and polar (Ser, Thr, Asn, Gln, His) residues appear to be much less conserved in protein-protein interfaces in general.

### The extent of location of experimental hot spot residues in conserved residue clusters

Residues targeted for alanine scanning mutagenesis are distributed over all the residue classes and have a wide range of sequence conservation (Additional file 1, Table S3 and Figure S7). Functionally important residues in protein-protein interfaces are usually those that contribute significantly to the free energy of binding - mutations resulting in binding energy changes of ≥ 2 kcal/mol are called hot spots [29]. The identification of clusters of conserved residues is probably a good way of identifying functionally important regions of the interface because it is likely that a sizeable number of hot spots will reside within such clusters. A group of 26 diverse protein-protein interfaces for which experimental alanine scanning mutagenesis data are available have been taken (compiled in [39]) and the conserved residue clusters present in each of them have been identified. Then the location of the experimentally determined 'hot' residues (identified using different ΔΔG cutoffs) have been mapped onto the interface and the fraction of these residues occurring within the conserved residue clusters was found out (Additional file 1, Table S2). Three groups of residues were considered - those with experimental ΔΔG values of ≥ 1, ≥ 1.5 and ≥ 2 kcal/mol. Of the 196 interface residues that contribute ≥ 1 kcal/mol to the binding energy, 106 (54.1%) occur within these clusters of conserved residues. When further restricted to those interface residues contributing 1.5 kcal/mol (or greater) or 2 kcal/mol (or more), the fraction of these that could be located within the conserved clusters increased to 56.8% (83/146) and 57.9% (55/95), respectively.

### Conserved residue clustering to discriminate interface from other surface patches

**Figure 5:**
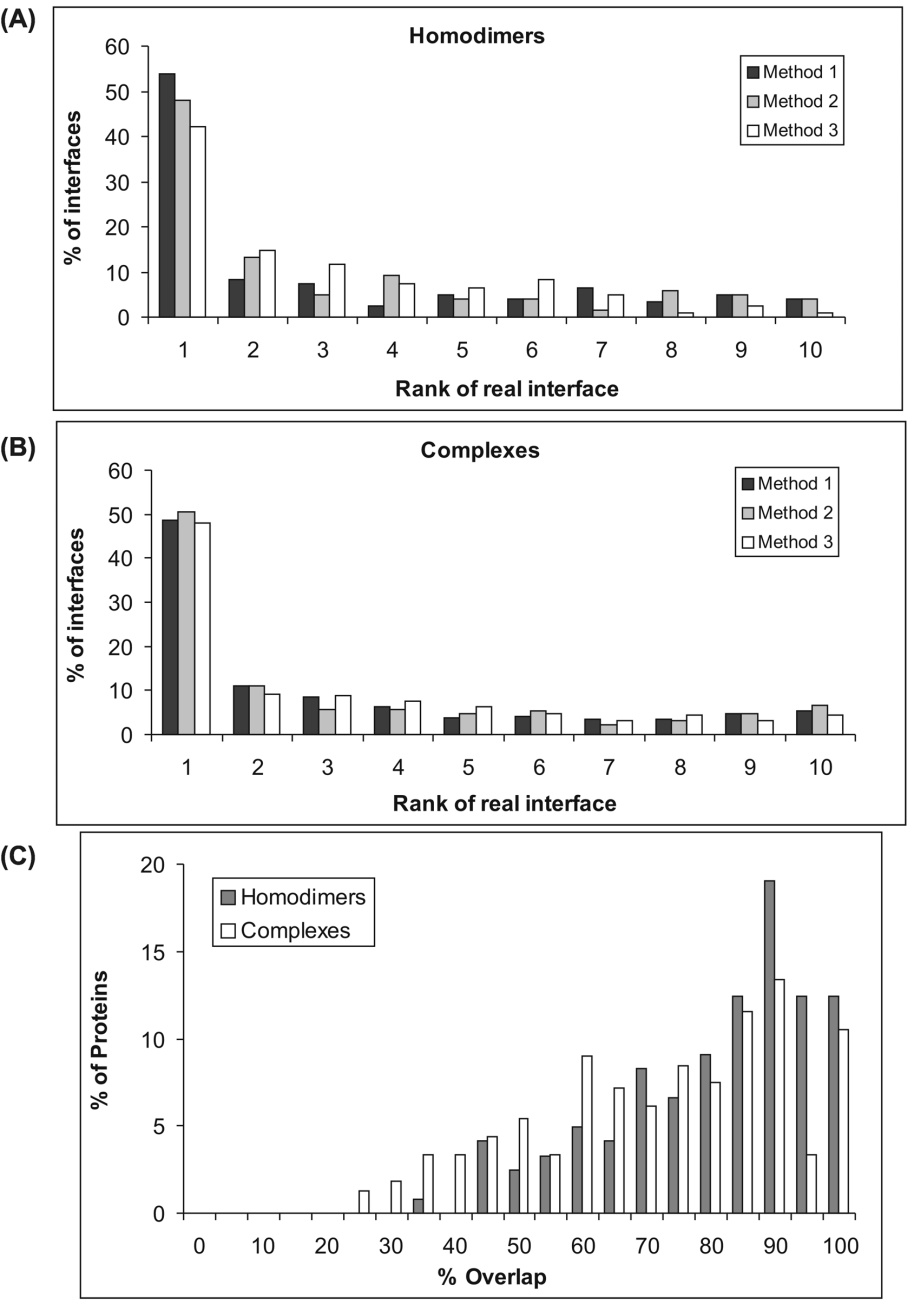
**Ranking of the interface relative to all possible surface patches**. Distribution of the degree of clustering of conserved residues within interfaces as compared to other surface patches for (A) homodimers, and (B) protein complexes. For each protein, the interface is ranked, relative to all other surface patches, as being in the top 10% (rank 1), 10-20% (rank 2), etc. according to the ρ value (Eq. 4). Methods 1-3 for generating the surface patches are described in Methods. (C) For each protein, the generated surface patch having the maximum overlap with the true interface is found out and the distribution of the % overlap is plotted for all proteins belonging to the two datasets.

**Figure 6:**
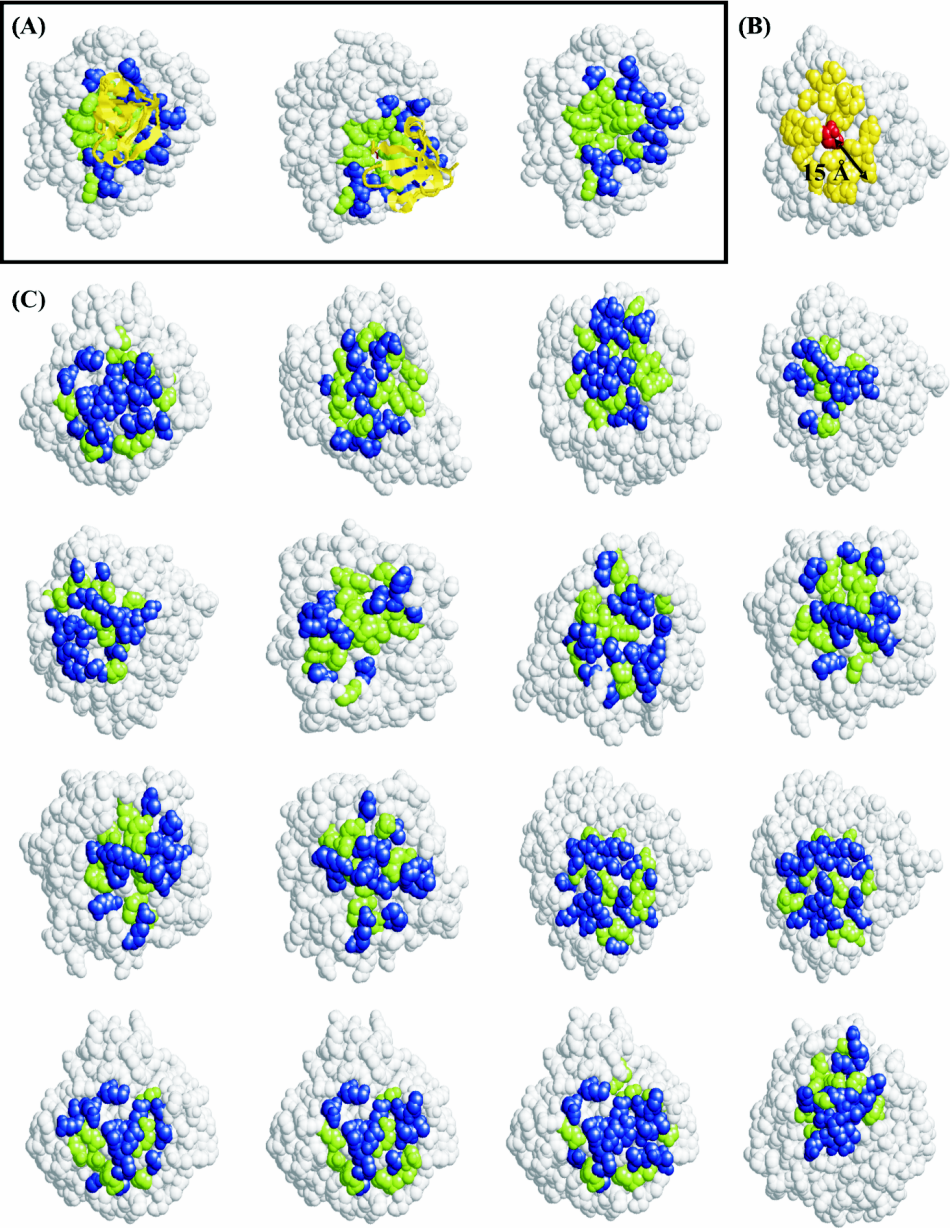
**Comparison of the clustering of conserved residues within the interface and other surface patches of human carboxypeptidase complexed to its inhibitor (PDB file, 1dtd)**. (A) The chain of interest (carboxypeptidase) is shown in spacefill (grey), its partner (inhibitor) in cartoon representation (yellow) in two different orientations. Conserved interface residues (on the enzyme) are colored green, the remaining interface residues are in blue. The partner protein is removed in the third view to clearly show the clustered nature of the conserved residues within the interface. (B) Diagram showing the construction of surface patches around each surface residue using a fixed cutoff of 15 Å (Method 1). (C) Sixteen different surface patches of the protein (in grey) are shown, in each of them the conserved residues (green) are scattered over the entire patch.

The clustering of conserved residues in the interface region can be compared to the clustering of conserved residues within alternative, randomly-collected surface patches on the protein. For each of the proteins studied a group of surface patches were created as described in Methods. For each of these surface patches, the clustering of conserved positions within them are compared to the residues comprising the entire patch. The ratio of clustering of the conserved positions relative to the overall patch (ρ) was then used to sort the surface patches (in descending order of ρ). A ranking of the true interface patch relative to all the other surface patches was then calculated (on a scale of 1 to 10). A rank of 1 indicates that the true interface is present in the top 10% of all surface patches and a rank of 10 indicates a location in the lowest 10% range in the distribution of ρ for all surface patches. Figure 5A,B assesses the extent to which this parameter can differentiate the interface region. Overall, in comparison to similar-size groups of surface patches, the clustering of conserved residues within the interface is more. All the three methods of generation of surface patches result in similar ranking. Of the 121 homodimeric interfaces, 25 (i.e., 20%) are ranked #1 (absolute #1 rather than rank bin 1) among random surface patches and 65 (53.7%) in the top 10% (i.e., rank 1) (Table 3). Of the complexes, 64 interfaces (16.5%) are ranked #1 among all random surface patches, and 189 (48.6%) in the top 10% bin. Figure 5C shows the distribution of the percentage of common residues (with the real interface) for the patch with maximum overlap (Eq. 5). Figure 6 and Additional file 1, Figure S8 illustrate examples of the clustering of conserved residues within the true interface as opposed to the distribution of conserved positions within randomly generated surface patches. In both these illustrations, the subset of conserved residues cluster together within the interface region, whereas in the other surface patches shown the conserved residues are distributed randomly all over the patch. The use of Eq. 1a instead of Eq. 1 (i.e., using background frequencies) does not improve the prediction accuracies (values given in square brackets in Table 3).

**Table 3:** Interface prediction accuracy, with heterocomplexes divided into functional classes

Interface type (number)	Number (and percentage) of interfaces with Rank 1		
	Method 1	Method 2	Method 3
Homodimers (121)	65/121 (53.7) [54/121 (45)]	58/121 (47.9)	51/121 (42.2)

Heterocomplexes (389)	189/389 (48.6) [180/389 (46.3)]	196/389 (50.4)	187/389 (48.1)

Enzyme-inhibitor (114)	77/114 (67.5)	80/114 (70.2)	75/114 (65.8)

Antibody-antigen (41)	10/41 (24.4)	09/41 (22.0)	10/41 (24.4)

Signaling complexes (78)	32/78 (41.0)	34/78 (43.6)	32/78 (41.0)

Others (156)	70/156 (44.9)	73/156 (46.8)	70/156 (44.9)

Details of the slightly different methods used to generate the surface patches are provided in Methods. Eq. 1 was used to calculate sequence entropy; however, in a few cases (values in square brackets) calculations were also repeated using Eq. 1a.

To study if the results depend on the nature of the complex we made a functional classification of heterocomplexes as interfaces belonging to enzyme-inhibitor, antigen-antibody, signaling complexes, and Others. For each of these four types, we found out the prediction accuracy separately (Table 3). In all three methods for generating surface patches, we find that the enzyme-inhibitor interfaces are predicted to a much higher degree of success compared to the other interface classes. Prediction accuracy of interfaces in antibody-antigen complexes is the lowest. This might reflect the fact that antibody sequences diverge quickly in order to recognize a wide repertoire of antigens, and therefore, any analysis based on conservation may not be appropriate while dealing with these complexes. Indeed, the statistics in Table 1 show that the general observation that conserved residues are clustered within the interface region does not seem to be the case for antibody-antigen interfaces.

We further examined the statistical significance of the degree of clustering of conserved residues within true interfaces as compared to that in random regions of the protein surface. The Z test was used for this purpose, defined as Z = (ρ_int _- < ρ>)/σ, where ρ_int _is the value (Eq. 4) for the real interface, and < ρ> is the average vale for all surface patches in the protein, σ being the standard deviation. For the homodimers, about 40% (49/121) interfaces contain conserved residues which are significantly more clustered compared to conserved residues present within other surface patches (Z > 1.64, the critical Z-score, corresponding to the 95^th ^percentile of the normal distribution). For the complexes, such significant clustering of conserved residues within the interface was observed in 38% (148/389) cases. Hence, for these interfaces, the clustered nature of the conserved residues alone is sufficient to distinguish the true interface from remaining surface patches.

## Discussion and Conclusions

This work investigates the degree of spatial clustering of conserved residues within protein-protein interfaces. Three main issues are addressed: (1) the distribution of conserved residues in interfaces, (2) the degree of overlap between the subset of conserved residue positions and experimentally determined binding hot spots, and, (3) the prediction of the interface using the distribution of conserved residues.

### Clustering of conserved residues within interfaces

A ρ value of > 1.0 indicating the clustering of conserved residues relative to all the residues in the interface (M_s,cons _> M_s,int_) can be seen in Figures 1, 2, Additional file 1, Figure S2 and Table 1. The clustering of conserved residues within protein-protein interfaces has an important implication - the identification of protein-protein binding sites may be facilitated by analyzing the clustering of conserved residues within all surface patches. The veracity of the conclusion that conserved residues in the interface tend to be spatially clustered has also been confirmed using yet another dataset - the Protein-protein Docking Benchmark 3.0 [38] (Additional file 1, Figure S9). Functionally important residues are almost always conserved throughout evolutionary history so as to preserve the integrity of biological interactions occurring in signaling and reaction pathways. These residues also need to act in tandem with one another which necessitates them to be located in close juxtaposition within protein structures and interfaces. The conserved residues prefer to be clustered with other neighboring conserved residues rather than be in isolation (less than 7.5% of conserved residues in both homodimer and heteroprotein interfaces occur as isolated conserved residues, Additional file 1, Figure S6). Overall, 52 (± 15) and 46 (± 21)% of the interface area in homodimers and complexes, respectively, are occupied by the conserved residues. The identification of conserved residues is based on multiple sequence alignments available at the HSSP database [36], with the sequence identities for the aligned sequences being in the range 30-100%. Although sometimes there might exist some variability in the position of binding sites in large protein families, it has been shown that close homologues (30-40% or higher sequence identity) almost invariably interact the same way [44]. As such the interface residues in one member of the multiple sequence alignment are likely to be part of the interface in all the other homologues as well.

Enzymes often have multiple clusters of conserved residues in the structural scaffold as well as in the protein-protein interface [22]. This is consistent with our observation that larger interfaces often have multiple clusters in the interface. Examples of interfaces with multiple clusters of conserved residues in the interface are provided in Additional file 1, Figure S5. The increasing number of distinct interface clusters with increasing interface size may reflect the fact that larger interfaces are often functionally more complex. For example, larger interfaces often consist of multiple patches contributed by different structural domains of the protein [35] and each of these domains contains a conserved interface cluster (as for example in Additional file 1, Figure S5B-D). The multiple clusters may be important for stabilizing the interaction in case of larger interfaces by forming distinct binding units (or "hot regions") which are characterized by cooperative interactions such as hydrogen bonding and salt bridges [30]. Each of the independent clusters probably contributes additively to the binding free energy. Hence the findings of this study appears to confirm the view of protein-protein interfaces as being locally optimized and consisting of well-packed sub-regions containing conserved and energetically important residues that form a network of interactions.

Experimental approaches for the identification of functionally important residues on protein surface involve mutagenesis of a large number of residues and recoding the change in activity or binding to other proteins. However, considering the large size of the protein-protein interfaces and without *a priori *knowledge of the binding site, such determination is time-consuming and fraught with technical difficulties. Therefore, computational efforts have been used to identify and target those regions likely to contain functionally important residues. For example, the evolutionary trace method (ET) [20] searches for spatial clusters of conserved residues and then maps them onto a representative three-dimensional structure to suggest probable functionally important sites. Landgraf *et al*. [7] also combined the structural environment and evolutionary variation of residues to detect functionally important residue clusters. A scoring scheme that did not take three-dimensional information into account performed poorly compared to their 3-D cluster analysis. Thus, spatial contiguity along with sequence conservation is important for inferring functionally relevant residue clusters. Even within protein structures and on protein surfaces, such structural clusters of evolutionary trace residues occur quite commonly and are found to be statistically significant [22,23,25,26,45,46]. These clusters almost consistently overlap with known functional sites of the protein surface and the potential of this sort of method for functional annotation from a structural genomics point of view is enormous [19,23,24,47]. The formation and use of interacting residue clusters within protein-DNA interfaces has also been observed as well [22,48] and the phenomenon is apparently universal to most, if not all, types of macromolecular recognition.

### Conserved residue clusters and energetically 'hot' regions in the interface

It is known that interface hot spot residues form clusters within densely packed 'hot regions', where they form networks of interactions contributing cooperatively to the stability of the complex [30]. Therefore, the degree of overlap between the conserved residue clusters and experimental hot spots has also been investigated in this work (overall results are shown in Additional file 1, Table S2). Although the observed correlation between our conserved interface clusters and experimental hot spots is moderate (~60% of hot spot residues can be localized to these clusters), the method has potential to identify and target mutagenesis experiments to appropriate sites. Availability of a larger group of experimental mutants may possibly increase the extent of this overlap. At the same time, however, it is also true that many binding energy hot spots do not actually contribute directly to the interface [49]. For example, some of them function by serving to orient other residues that are directly involved, for instance in hydrogen bonding networks within the interface. Of the 20 amino acid residues, hydrophobic and aromatic groups seem to be among the most preferred in conserved clusters (Figure 4), and these are the same residues that are preferred in the interface core [43]. Gly seems to be an exception in that it is preferred in conserved residue clusters, but not in the core. Indeed because of its small size Gly can preferentially couple with many other residue types and has a higher level of conservation [50]. Although conserved polar residues (Arg, Gln, His, Asp and Asn) are known to constitute hot spots [51], these are not prominent in the conserved subset relative to the overall interface. Fewer in number they may still confer specificity to the interaction (by participating in critical hydrogen bonds or salt bridges) [39]. The finding that conserved Trp residues (and to a lesser extent Phe and Met) on the protein surface indicate likely binding sites [52], is also supported by the high propensity of these residues to be observed in the conserved region of the interface (Figure 4), along with the general low level of occurrence, especially of Trp and Met, in proteins. It has also been shown that the majority of the conserved residues in the binding region overlap clusters of high-frequency vibrating residues [53].

### Clustering of conserved residues for the prediction of binding site

We investigated the potential use of the clustering of conserved residues for the identification of the binding site by comparing this feature in the real interface against all other surface patches (Figure 5). In about 50% of cases in both datasets, the real interface region is listed in the top 10% (rank #1) of all surface patches, actually occupying the top position (absolute #1) in 16-20% cases. Jones and Thornton [41] have previously characterized protein interaction sites in complexes of known structures using six parameters (solvation potential, residue interface propensity, hydrophobicity, planarity, protrusion and accessible surface area) to evaluate what differentiates them from other surface patches on the protein surface. Although none of the parameters were definitive, the majority showed trends for the observed interface to be distinguished from other surface patches. Furthermore, a combined score (using these six parameters) giving the probability of a surface patch forming protein-protein interactions was also put forward giving a success rate of 66% for 59 structures [54]. Thus there is a scope for combining evolutionary and physicochemical features for identifying the binding sites.

A question may be asked if a direct assessment by first identifying conserved residues on the protein surface and then searching for spatial clusters could have been performed (instead of dividing the protein surface into patches similar in size to the interface, and then searching for conserved residues). Methods like the Evolutionary Trace (ET) [20] use the former approach - they first locate completely conserved and class-specific (i.e., conserved within sub-groups) residues and then check if these residues form spatial clusters on the protein surface. Such a direct assessment of conserved residue clusters is likely to yield significant results when the functional sites being identified are highly conserved and extremely crucial to the protein's function, for example enzyme active sites. However, protein-protein interfaces are extensive, involving a much larger number of residues, which are less conserved in general than enzyme active sites or other small-molecule binding sites. In many cases the same protein may exist in equilibrium between different oligomeric forms, such that the interface in one form may be surface exposed in another [55]. As such we had to use a less stringent condition for the definition of conserved residues, and compare the clustering of such residues relative to the entire interface (or surface patches of similar size) rather than using a direct assessment of the distribution of conserved residues over the whole surface (as done in ET).

### Comparison with machine learning techniques

Recently, machine learning techniques, such as Support Vector Machines and Neural Networks have also incorporated the use of sequence conservation metrics to enhance the likelihood of predicting which surface residues of a given protein form an interface [56-58]. In one of the earlier applications of the SVM-based approach incorporating evolutionary information as an additional attribute, the prediction accuracy for the classification of interface residues reached 64% [56]. However, when classifiers based on only evolution were used the value was lower (54%). This is comparable to the value for the percentage (~50%) of interfaces that are ranked 1 among all surface patches (Figure 5). This study, however, does the prediction from sequence unlike the present work where we use the crystal structure to define surface patches and then score them for the likelihood of being a binding interface. In another study which starts from the protein structure for surface patch generation, a combination of 7 properties, including residue conservation, was used to predict protein binding sites and achieved a maximum prediction accuracy of 76%, 64% being the value for enzyme-inhibitor complexes [57]. Interestingly, we obtain a comparable prediction success (~70%) on the enzyme-inhibitor complexes using just a single parameter (conserved residue clustering) (Table 3). That evolutionary conservation has a greater discriminatory power for the identification of interface residues has also been shown [58]; however, there was no consideration of any clustering. In another study, 52% of 'precisely' identified and 77% of 'correctly' predicted binding sites were reported in a study that trained an SVM classifier using structural conservation scores as one of the parameters [59]. Though the authors noted that the structurally conserved residues were more clustered in interface regions compared to the non-interface surface, the concept of clustering of conserved residues was not directly used to train the SVM classifier. ProMate is a program to predict protein-protein interfaces using an optimized combination of 9 different metrics including evolutionary conservation - 70% success rate (on 51 protein structures) has been reported [60]. ProMate has also been combined with another prediction program based on surface conservation and structural information (WHISCY) [61]. The algorithm implemented in WHISCY uses a sequence alignment to calculate a prediction score (the residue is predicted as "interface" if the score exceeds a certain threshold) for each surface residue of the test protein. It also recognizes that predicted interface residues that are surrounded by other predicted interface residues are more likely to be part of the actual binding site rather than isolated predicted residues. To incorporate this observation, the scores for all the surface residues are taken and smoothed over the surface of the protein structure. This "smoothing" ensures that the scores of the spatial neighbors on the surface are also taken into account. When the high-scoring residues are visualized on the structure, they are often found clustered. However, what we propose here is a scheme to explicitly measure the degree of clustering of conserved residues within a surface patch and use that for the prediction. Lastly, neural network techniques are also available for the prediction of protein-protein interaction sites and may achieve a success of 70-80% [62,63]. Although objective comparison between all these algorithms is difficult as each study used different interface definitions and criteria for success in addition to using different datasets, it does appear that the identification of conserved residues and their spatial clustering offers a convenient way to locate the binding site. To conclude, residue conservation has been a useful metric for many prediction algorithms. The incorporation of the clustering procedure enumerated here should improve the performance of these methods.

## Authors' contributions

PC conceptualized the work that was carried out by MG. MG and PC participated in interpretation of the data and writing the manuscript. Both the authors have read and accepted the final version of the manuscript.

## Supplementary Material

Additional file 1 **The file contains three tables (numbered S1 to S3), and nine figures (numbered S1 to S9)**.
Table S1. Values of the parameters indicating the clustering of conserved residues in individual interfaces.
Table S2. Location of experimental hot spots within the conserved residue clusters in the interface.
Table S3. Distribution of 462 alanine scanned interface residues among the seven residue classes.
Figure S1. Representative examples of interfaces showing the clustered nature of evolutionarily conserved residues.
Figure S2. Plots of Ms,cons versus Ms,int.
Figure S3. Plots of M_s,cons _versus < M_s,random_>.
Figure S4. Number of interface residues and conserved residues as a function of interface area.
Figure S5. Multiple clusters of evolutionary conserved residues in protein interfaces.
Figure S6. Distribution of cluster size.
Figure S7. The level of sequence conservation of residues subjected to alanine scanning experiments.
Figure S8. Comparison of the clustering of conserved residues within the subunit interface and other surface patches.
Figure S9. Plot of Ms,cons versus Ms,int for interfaces from the bound forms 124 protein complexes described in the Protein-protein Docking Benchmark version 3.0. Click here for file
