# Supplementary material for: Conserved residue clusters at protein-protein interfaces and their use in binding site identification
Source: BMC Bioinformatics. 2010 May 27;11:286. doi: 10.1186/1471-2105-11-286 (PMC2894039; doi:10.1186/1471-2105-11-286)

**Supplementary material**

Table S1. Values of the parameters indicating the clustering of conserved residues in individual interfaces

(A) Homodimers

| PDB code | <s>_int_^a^  (# aligned sequences) | #  interface, conserved residues | M_s_ | | ρ | # sub-clusters |
| --- | --- | --- | --- | --- | --- | --- |
|  |  |  | Conserved residues | All interface residues |  |  |
| 12as | 0.55 (160) | 52, 26 | 0.071 | 0.065 | 1.09 | 2 |
| 1a3c | 0.70 (891) | 27, 17 | 0.099 | 0.089 | 1.11 | 1 |
| 1a4i | 0.91 (2817) | 38, 18 | 0.092 | 0.079 | 1.17 | 1 |
| 1a4u | 0.52 (596) | 63, 27 | 0.066 | 0.064 | 1.03 | 1 |
| 1aa7 | 0.001 (3000) | 26, 23 | 0.088 | 0.090 | 0.98 | 1 |
| 1ad3 | 1.05 (719) | 110, 48 | 0.057 | 0.046 | 1.25 | 5 |
| 1ade | 0.81 (921) | 78, 41 | 0.070 | 0.058 | 1.21 | 1 |
| 1af5 | 0.73 (30) | 24, 12 | 0.120 | 0.090 | 1.33 | 1 |
| 1afw | 1.08 (4432) | 70, 31 | 0.067 | 0.061 | 1.10 | 3 |
| 1ajs | 0.81 (1685) | 99, 49 | 0.061 | 0.051 | 1.19 | 3 |
| 1amk | 0.70 (1456) | 39, 22 | 0.088 | 0.081 | 1.09 | 1 |
| 1aor | 1.42 (203) | 33, 14 | 0.086 | 0.080 | 1.07 | 1 |
| 1aq6 | 1.16 (205) | 54, 28 | 0.076 | 0.066 | 1.15 | 2 |
| 1auo | 0.90 (246) | 20, 9 | 0.094 | 0.089 | 1.05 | 1 |
| 1b3a | 0.85 (412) | 23, 10 | 0.107 | 0.095 | 1.12 | 2 |
| 1b5e | 0.49 (11) | 65, 30 | 0.070 | 0.064 | 1.09 | 2 |
| 1b67 | 0.58 (250) | 40, 20 | 0.075 | 0.073 | 1.03 | 2 |
| 1b8a | 0.89 (1188) | 114, 55 | 0.052 | 0.048 | 1.08 | 3 |
| 1b8j | 1.32 (868) | 103, 42 | 0.066 | 0.053 | 1.24 | 3 |
| 1bam | 0.24 (8) | 18, 13 | 0.109 | 0.097 | 1.12 | 1 |
| 1bbh | 0.96 (30) | 24, 10 | 0.100 | 0.097 | 1.03 | 1 |
| 1bd0 | 0.81 (1533) | 85, 45 | 0.057 | 0.052 | 1.10 | 3 |
| 1bif | 0.91 (327) | 28, 15 | 0.081 | 0.069 | 1.16 | 2 |
| 1biq | 0.40 (341) | 74, 45 | 0.063 | 0.055 | 1.14 | 2 |
| 1bis | 0.015 (4500) | 40, 32 | 0.076 | 0.073 | 1.04 | 1 |
| 1bjw | 0.95 (1408) | 76, 35 | 0.060 | 0.057 | 1.05 | 3 |
| 1bkp | 0.80 (768) | 58, 29 | 0.069 | 0.062 | 1.11 | 1 |
| 1bmd | 0.63 (395) | 41, 23 | 0.079 | 0.069 | 1.14 | 2 |
| 1brw | 0.76 (383) | 27, 16 | 0.101 | 0.085 | 1.18 | 1 |
| 1bsl | 0.37 (221) | 55, 32 | 0.075 | 0.070 | 1.08 | 1 |
| 1bsr | 0.82 (740) | 48, 22 | 0.083 | 0.069 | 1.21 | 2 |
| 1buo | 0.95 (457) | 49, 24 | 0.071 | 0.064 | 1.12 | 2 |
| 1bxg | 1.15 (583) | 25, 11 | 0.093 | 0.085 | 1.09 | 1 |
| 1bxk | 0.89 (3994) | 38, 20 | 0.092 | 0.077 | 1.20 | 1 |
| 1cdc | 0.67 (34) | 85, 39 | 0.057 | 0.056 | 1.01 | 3 |
| 1cg2 | 0.74 (58) | 40, 24 | 0.083 | 0.076 | 1.10 | 2 |
| 1chm | 0.35 (56) | 85, 56 | 0.056 | 0.052 | 1.07 | 4 |
| 1cmb | 0.04 (105) | 39, 34 | 0.084 | 0.081 | 1.04 | 1 |
| 1cnz | 0.95 (1411) | 63, 28 | 0.071 | 0.058 | 1.22 | 2 |
| 1coz | 0.86 (589) | 24, 12 | 0.091 | 0.083 | 1.09 | 1 |
| 1csh | 0.72 (349) | 117, 60 | 0.059 | 0.051 | 1.17 | 4 |
| 1ctt | 0.67 (444) | 53, 27 | 0.084 | 0.070 | 1.19 | 1 |
| 1cvu | 0.51 (246) | 69, 45 | 0.063 | 0.056 | 1.13 | 3 |
| 1czj | 1.03 (29) | 20, 7 | 0.112 | 0.094 | 1.19 | 1 |
| 1daa | 0.94 (460) | 61, 32 | 0.085 | 0.068 | 1.24 | 1 |
| 1dor | 0.87 (217) | 60, 28 | 0.068 | 0.062 | 1.10 | 2 |
| 1dpg | 0.92 (1102) | 62, 28 | 0.077 | 0.062 | 1.24 | 2 |
| 1dqs | 0.88 (3152) | 49, 22 | 0.095 | 0.068 | 1.40 | 1 |
| 1dxg | 0.52 (50) | 21, 11 | 0.116 | 0.107 | 1.08 | 1 |
| 1e98 | 1.06 (188) | 21, 10 | 0.114 | 0.089 | 1.28 | 1 |
| 1ebh | 0.53 (1483) | 55, 34 | 0.073 | 0.065 | 1.14 | 1 |
| 1f13 | 0.89 (310) | 83, 35 | 0.057 | 0.047 | 1.20 | 4 |
| 1fip | 0.72 (346) | 43, 23 | 0.084 | 0.075 | 1.12 | 1 |
| 1fro | 0.90 (617) | 97, 46 | 0.070 | 0.055 | 1.26 | 2 |
| 1gvp | 0.62 (18) | 24, 9 | 0.102 | 0.092 | 1.11 | 1 |
| 1hhp | 0.001 (1500) | 36, 32 | 0.080 | 0.079 | 1.01 | 2 |
| 1hjr | 0.73 (560) | 26, 15 | 0.101 | 0.087 | 1.15 | 1 |
| 1hss | 0.27 (1280) | 31, 16 | 0.088 | 0.081 | 1.09 | 2 |
| 1hxp | 0.62 (774) | 87, 51 | 0.057 | 0.053 | 1.08 | 3 |
| 1icw | 0.92 (259) | 28, 12 | 0.103 | 0.086 | 1.19 | 1 |
| 1imb | 1.07 (1262) | 44, 24 | 0.082 | 0.072 | 1.13 | 1 |
| 1isa | 0.42 (1500) | 23, 14 | 0.099 | 0.090 | 1.09 | 1 |
| 1ivy | 1.30 (1123) | 49, 22 | 0.080 | 0.065 | 1.23 | 2 |
| 1jhg | 0.81 (109) | 53, 24 | 0.085 | 0.069 | 1.22 | 1 |
| 1jsg | 0.60 (11) | 21, 8 | 0.101 | 0.092 | 1.09 | 1 |
| 1kba | 0.88 (432) | 14, 5 | 0.135 | 0.107 | 1.26 | 1 |
| 1kpf | 1.10 (981) | 46, 22 | 0.090 | 0.075 | 1.20 | 1 |
| 1lyn | 0.34 (31) | 24, 15 | 0.099 | 0.089 | 1.11 | 1 |
| 1m6p | 0.76 (78) | 34, 17 | 0.082 | 0.078 | 1.05 | 1 |
| 1mkb | 0.56 (343) | 43, 24 | 0.086 | 0.072 | 1.18 | 1 |
| 1mor | 0.57 (986) | 69, 40 | 0.063 | 0.058 | 1.09 | 3 |
| 1nox | 1.10 (268) | 75, 34 | 0.067 | 0.058 | 1.16 | 2 |
| 1nse | 0.74 (368) | 75, 39 | 0.071 | 0.059 | 1.21 | 2 |
| 1nsy | 0.95 (398) | 64, 31 | 0.074 | 0.058 | 1.27 | 1 |
| 1oac | 0.95 (185) | 192, 86 | 0.044 | 0.039 | 1.11 | 5 |
| 1opy | 0.69 (41) | 30, 18 | 0.102 | 0.088 | 1.16 | 1 |
| 1pgt | 0.88 (445) | 32, 13 | 0.081 | 0.077 | 1.06 | 1 |
| 1pre | 0.56 (405) | 80, 41 | 0.043 | 0.042 | 1.02 | 5 |
| 1qfh | 0.75 (10) | 58, 28 | 0.064 | 0.060 | 1.07 | 3 |
| 1qhi | 0.61 (770) | 48, 23 | 0.068 | 0.070 | 0.98 | 2 |
| 1qr2 | 0.997 (326) | 54, 26 | 0.082 | 0.064 | 1.28 | 1 |
| 1r2f | 0.64 (460) | 50, 24 | 0.069 | 0.065 | 1.06 | 2 |
| 1reg | 1.15 (46) | 18, 5 | 0.135 | 0.112 | 1.21 | 1 |
| 1rfb | 0.46 (127) | 73, 40 | 0.061 | 0.058 | 1.06 | 2 |
| 1rpo | 0.35 (36) | 35, 22 | 0.082 | 0.076 | 1.08 | 1 |
| 1ses | 0.77 (882) | 59, 28 | 0.074 | 0.066 | 1.13 | 1 |
| 1slt | 0.86 (254) | 16, 8 | 0.110 | 0.102 | 1.08 | 1 |
| 1smn | 1.19 (100) | 27, 10 | 0.095 | 0.095 | 1.00 | 1 |
| 1smt | 1.19 (1920) | 49, 21 | 0.085 | 0.070 | 1.22 | 2 |
| 1sox | 1.14 (1549) | 47, 19 | 0.084 | 0.072 | 1.17 | 2 |
| 1tc1 | 0.90 (1824) | 39, 20 | 0.082 | 0.071 | 1.15 | 1 |
| 1tox | 0.01 (60) | 119, 116 | 0.048 | 0.048 | 1.00 | 4 |
| 1trk | 0.71 (1349) | 118, 64 | 0.056 | 0.046 | 1.22 | 5 |
| 1uby | 0.85 (195) | 53, 27 | 0.070 | 0.064 | 1.10 | 1 |
| 1utg | 0.28 (13) | 40, 23 | 0.084 | 0.076 | 1.10 | 1 |
| 1vfr | 0.81 (184) | 91, 43 | 0.064 | 0.055 | 1.15 | 2 |
| 1vok | 0.58 (603) | 47, 29 | 0.080 | 0.066 | 1.22 | 1 |
| 1wtl | 1.06 (1594) | 21, 8 | 0.103 | 0.094 | 1.10 | 1 |
| 1xso | 0.78 (1790) | 21, 12 | 0.123 | 0.102 | 1.21 | 1 |
| 2arc | 0.48 (78) | 18, 10 | 0.099 | 0.093 | 1.07 | 1 |
| 2ccy | 0.16 (3) | 20, 15 | 0.099 | 0.094 | 1.05 | 1 |
| 2hdh | 0.94 (2840) | 40, 19 | 0.084 | 0.072 | 1.18 | 1 |
| 2ilk | 0.31 (132) | 96, 57 | 0.054 | 0.052 | 1.06 | 2 |
| 2lig | 0.51 (61) | 47, 19 | 0.064 | 0.058 | 1.12 | 2 |
| 2mcg | 1.09 (1542) | 57, 24 | 0.065 | 0.056 | 1.15 | 3 |
| 2nac | 0.92 (235) | 98, 48 | 0.064 | 0.056 | 1.13 | 2 |
| 2ohx | 0.79 (1500) | 49, 28 | 0.077 | 0.070 | 1.09 | 1 |
| 2spc | 0.60 (53) | 60, 36 | 0.051 | 0.049 | 1.03 | 1 |
| 2sqc | 1.19 (181) | 23, 9 | 0.100 | 0.092 | 1.09 | 1 |
| 2tct | 0.75 (187) | 63, 32 | 0.062 | 0.058 | 1.06 | 3 |
| 2tgi | 0.70 (721) | 37, 18 | 0.087 | 0.074 | 1.17 | 2 |
| 3dap | 0.60 (31) | 71, 37 | 0.072 | 0.063 | 1.15 | 1 |
| 3grs | 0.88 (894) | 87, 47 | 0.063 | 0.055 | 1.15 | 3 |
| 3sdh | 0.68 (21) | 25, 13 | 0.092 | 0.083 | 1.11 | 1 |
| 3ssi | 0.62 (41) | 31, 17 | 0.103 | 0.091 | 1.13 | 1 |
| 4cha | 1.17 (3000) | 33, 12 | 0.100 | 0.075 | 1.34 | 1 |
| 4kbp | 0.72 (270) | 42, 18 | 0.083 | 0.069 | 1.20 | 1 |
| 5csm | 0.69 (107) | 57, 28 | 0.061 | 0.058 | 1.05 | 3 |
| 5rub | 0.26 (4905) | 82, 48 | 0.059 | 0.059 | 1.00 | 3 |
| 8prk | 1.14 (409) | 23, 9 | 0.095 | 0.085 | 1.12 | 1 |
| 9wga | 1.32 (1290) | 67, 22 | 0.067 | 0.066 | 1.01 | 2 |

(B) Protein-protein complexes

| PDB code | <s>_int_^a^  (# aligned sequences) | #  interface, conserved residues | M_s_ | | ρ | # sub-clusters |
| --- | --- | --- | --- | --- | --- | --- |
|  |  |  | Conserved residues | All interface residues |  |  |
| 1a2kA | 0.62 (2962) | 26, 15 | 0.106 | 0.096 | 1.09 | 2 |
| 1a2kD | 0.54 | 22, 14 | 0.112 | 0.105 | 1.06 | 1 |
| 1a2yA | 1.41 (1754) | 20, 7 | 0.095 | 0.093 | 1.01 | 2 |
| 1a2yC | 1.16 | 21, 6 | 0.100 | 0.100 | 1.00 | 1 |
| 1acbE | 0.71 (2909) | 31, 16 | 0.124 | 0.101 | 1.23 | 1 |
| 1acbI | 0.58 | 18, 10 | 0.133 | 0.111 | 1.19 | 1 |
| 1avaA | 0.87 (434) | 40, 19 | 0.078 | 0.070 | 1.11 | 2 |
| 1avaC | 1.09 | 39, 17 | 0.090 | 0.073 | 1.24 | 2 |
| 1avwA | 0.85 (1450) | 36, 19 | 0.127 | 0.093 | 1.36 | 1 |
| 1avwB | 1.06 | 19, 6 | 0.139 | 0.099 | 1.40 | 2 |
| 1axiA | 0.46 (1518) | 36, 18 | 0.080 | 0.073 | 1.09 | 3 |
| 1axiB | 0.77 | 35, 16 | 0.081 | 0.076 | 1.07 | 3 |
| 1ay7A | 0.45 (109) | 20, 11 | 0.125 | 0.106 | 1.18 | 1 |
| 1ay7B | 0.48 | 17, 10 | 0.131 | 0.109 | 1.20 | 1 |
| 1azzA | 1.01 (2988) | 32, 14 | 0.127 | 0.091 | 1.40 | 1 |
| 1azzC | 0.76 | 24, 14 | 0.116 | 0.103 | 1.13 | 2 |
| 1b0nA | 0.58 (984) | 38, 23 | 0.091 | 0.082 | 1.12 | 1 |
| 1b0nB | 0.17 | 27, 20 | 0.090 | 0.092 | 0.98 | 1 |
| 1bj1H | 1.46 (3650) | 22, 8 | 0.095 | 0.100 | 0.96 | 3 |
| 1bj1W | 1.03 | 18, 7 | 0.104 | 0.112 | 0.93 | 1 |
| 1blxA | 0.94 (1206) | 29, 13 | 0.101 | 0.090 | 1.13 | 1 |
| 1blxB | 1.39 | 35, 15 | 0.090 | 0.085 | 1.06 | 2 |
| 1brsA | 0.60 (180) | 23, 12 | 0.112 | 0.096 | 1.16 | 1 |
| 1brsD | 0.46 | 20, 12 | 0.121 | 0.107 | 1.12 | 1 |
| 1bthH | 1.02 (2828) | 42, 18 | 0.124 | 0.086 | 1.43 | 1 |
| 1bthP | 0.91 | 25, 12 | 0.127 | 0.106 | 1.21 | 1 |
| 1c1yA | 1.03 (1499) | 21, 9 | 0.101 | 0.083 | 1.21 | 1 |
| 1c1yB | 0.26 | 18, 12 | 0.097 | 0.093 | 1.05 | 1 |
| 1ca0B | 0.71 (4612) | 29, 17 | 0.116 | 0.104 | 1.12 | 1 |
| 1ca0D | 0.90 | 15, 6 | 0.195 | 0.143 | 1.37 | 1 |
| 1cgiE | 0.97 (1324) | 36, 17 | 0.116 | 0.090 | 1.29 | 1 |
| 1cgiI | 1.24 | 21, 7 | 0.113 | 0.100 | 1.13 | 1 |
| 1choE | 0.0 (2474) | 30, 0 | - | 0.095 | - | - |
| 1choI | 0.77 | 15, 6 | 0.146 | 0.117 | 1.24 | 1 |
| 1clvA | 0.47 (727) | 39, 24 | 0.095 | 0.083 | 1.15 | 1 |
| 1clvI | 0.0 | 23, 0 | - | 0.105 | - | - |
| 1cseE | 0.77 (1460) | 30, 16 | 0.120 | 0.101 | 1.19 | 1 |
| 1cseI | 0.60 | 15, 9 | 0.132 | 0.114 | 1.16 | 1 |
| 1cxzA | 1.19 (1477) | 24, 8 | 0.088 | 0.086 | 1.02 | 2 |
| 1cxzB | 0.04 | 23, 21 | 0.082 | 0.083 | 0.99 | 2 |
| 1czyA | 0.90 (95) | 23, 8 | 0.112 | 0.108 | 1.04 | 1 |
| 1czyD | 0.00 | 7, 0 | - | 0.135 | - | - |
| 1d4vA | 1.40 (281) | 19, 5 | 0.108 | 0.078 | 1.38 | 2 |
| 1d4vB | 1.30 | 21, 9 | 0.095 | 0.091 | 1.05 | 2 |
| 1danL | 1.28 (1408) | 39, 13 | 0.064 | 0.056 | 1.15 | 4 |
| 1danT | 0.86 | 54, 22 | 0.061 | 0.061 | 1.00 | 4 |
| 1df9A | 0.37 (710) | 45, 14 | 0.110 | 0.065 | 1.71 | 1 |
| 1df9C | 0.58 | 29, 15 | 0.076 | 0.074 | 1.03 | 2 |
| 1dfjE | 0.90 (500) | 40, 16 | 0.099 | 0.076 | 1.31 | 2 |
| 1dfjI | 1.40 | 50, 19 | 0.076 | 0.065 | 1.18 | 3 |
| 1dhkA | 0.53 (1516) | 51, 28 | 0.082 | 0.070 | 1.17 | 2 |
| 1dhkB | 1.05 | 44, 21 | 0.092 | 0.079 | 1.17 | 3 |
| 1dkdA | 0.26 (1500) | 15, 10 | 0.120 | 0.114 | 1.05 | 1 |
| 1dkdE | 0.00 | 10, 0 | - | 0.137 | - | - |
| 1ds6A | 0.87 (1367) | 32, 16 | 0.102 | 0.083 | 1.23 | 1 |
| 1ds6B | 0.54 | 37, 21 | 0.087 | 0.077 | 1.13 | 2 |
| 1dtdA | 0.78 (296) | 29, 15 | 0.123 | 0.094 | 1.31 | 1 |
| 1dtdB | 0.00 | 19, 0 | - | 0.102 | - | - |
| 1dvfA | 1.48 (3550) | 26, 11 | 0.094 | 0.091 | 1.03 | 3 |
| 1dvfC | 1.49 | 25, 9 | 0.086 | 0.091 | 0.94 | 1 |
| 1dzbA | 1.40 (3716) | 28, 11 | 0.083 | 0.086 | 0.96 | 3 |
| 1dzbX | 1.16 | 22, 9 | 0.115 | 0.096 | 1.20 | 2 |
| 1e44A | 0.39 (28) | 34, 20 | 0.089 | 0.083 | 1.07 | 2 |
| 1e44B | 0.78 | 32, 14 | 0.085 | 0.082 | 1.03 | 2 |
| 1e96A | 0.96 (1246) | 18, 7 | 0.099 | 0.106 | 0.94 | 2 |
| 1e96B | 0.66 | 20, 10 | 0.103 | 0.099 | 1.04 | 2 |
| 1eayA | 1.12 (1504) | 16, 6 | 0.136 | 0.106 | 1.28 | 1 |
| 1eayC | 0.57 | 19, 11 | 0.100 | 0.097 | 1.04 | 1 |
| 1eerA | 0.36 (76) | 41, 19 | 0.077 | 0.068 | 1.12 | 4 |
| 1eerB | 0.21 | 53, 16 | 0.073 | 0.063 | 1.17 | 2 |
| 1efnA | 0.82 (2169) | 16, 8 | 0.132 | 0.111 | 1.20 | 1 |
| 1efnB | 0.06 | 17, 15 | 0.107 | 0.109 | 0.99 | 1 |
| 1efuA | 0.16 (2237) | 60, 44 | 0.066 | 0.062 | 1.08 | 4 |
| 1efuB | 0.51 | 48, 28 | 0.064 | 0.056 | 1.15 | 4 |
| 1emvA | 0.86 (111) | 24, 10 | 0.122 | 0.101 | 1.21 | 1 |
| 1emvB | 1.11 | 22, 9 | 0.117 | 0.103 | 1.14 | 1 |
| 1euvA | 0.74 (239) | 36, 17 | 0.102 | 0.081 | 1.25 | 2 |
| 1euvB | 0.71 | 25, 11 | 0.086 | 0.084 | 1.02 | 2 |
| 1ewyA | 0.93 (974) | 40, 13 | 0.097 | 0.060 | 1.62 | 1 |
| 1ewyC | 1.05 | 41, 17 | 0.091 | 0.075 | 1.21 | 3 |
| 1f34A | 1.11 (840) | 54, 22 | 0.109 | 0.074 | 1.48 | 2 |
| 1f34B | 0.35 | 43, 20 | 0.083 | 0.070 | 1.19 | 3 |
| 1finA | 0.89 (1303) | 52, 25 | 0.076 | 0.071 | 1.07 | 2 |
| 1finB | 0.83 | 42, 20 | 0.077 | 0.069 | 1.12 | 2 |
| 1fleE | 1.06 (1431) | 27, 10 | 0.141 | 0.092 | 1.53 | 1 |
| 1fleI | 1.12 | 20, 8 | 0.102 | 0.107 | 0.95 | 1 |
| 1fltW | 0.72 (420) | 17, 9 | 0.108 | 0.098 | 1.10 | 2 |
| 1fltX | 1.12 | 16, 7 | 0.116 | 0.097 | 1.20 | 1 |
| 1fnsA | 0.43 (1598) | 15, 8 | 0.104 | 0.111 | 0.93 | 1 |
| 1fnsH | 1.40 | 17, 6 | 0.088 | 0.113 | 0.78 | 1 |
| 1fs1A | 0.62 (648) | 18, 11 | 0.111 | 0.101 | 1.10 | 1 |
| 1fs1B | 0.53 | 24, 12 | 0.106 | 0.093 | 1.13 | 1 |
| 1fyhA | 0.74 (373) | 28, 14 | 0.080 | 0.084 | 0.96 | 3 |
| 1fyhB | 0.72 | 29, 12 | 0.078 | 0.079 | 0.98 | 2 |
| 1gg2A | 0.47 (1063) | 33, 21 | 0.079 | 0.070 | 1.13 | 2 |
| 1gg2B | 0.57 | 41, 19 | 0.071 | 0.070 | 1.01 | 2 |
| 1gl1A | 0.87 (8869) | 29, 14 | 0.121 | 0.095 | 1.28 | 1 |
| 1gl1I | 0.66 | 20, 8 | 0.118 | 0.105 | 1.13 | 1 |
| 1gl4A | 0.46 (303) | 28, 18 | 0.088 | 0.086 | 1.03 | 1 |
| 1gl4B | 1.35 | 24, 10 | 0.104 | 0.094 | 1.11 | 2 |
| 1gotA | 0.50 (1032) | 35, 22 | 0.078 | 0.069 | 1.13 | 2 |
| 1gotB | 0.59 | 41, 22 | 0.073 | 0.071 | 1.03 | 2 |
| 1guaA | 1.09 (1499) | 20, 10 | 0.105 | 0.094 | 1.12 | 1 |
| 1guaB | 0.28 | 17, 13 | 0.103 | 0.098 | 1.05 | 1 |
| 1h1rA | 0.91 (1302) | 47, 24 | 0.071 | 0.068 | 1.05 | 2 |
| 1h1rB | 0.79 | 43, 21 | 0.080 | 0.069 | 1.15 | 2 |
| 1h2kA | 0.46 (141) | 52, 25 | 0.067 | 0.069 | 0.96 | 4 |
| 1h2kS | 0.06 | 22, 19 | 0.081 | 0.082 | 0.99 | 3 |
| 1h2tC | 0.47 (571) | 53, 30 | 0.064 | 0.058 | 1.11 | 4 |
| 1h2tZ | 1.24 | 39, 15 | 0.070 | 0.070 | 1.00 | 2 |
| 1hiaA | 0.94 (5216) | 32, 15 | 0.123 | 0.091 | 1.35 | 1 |
| 1hiaI | 0.70 | 18, 10 | 0.112 | 0.105 | 1.07 | 1 |
| 1i7wC | 0.33 (1209) | 86, 63 | 0.049 | 0.043 | 1.14 | 4 |
| 1i7wD | 0.32 | 44, 28 | 0.046 | 0.047 | 0.99 | 5 |
| 1iarA | 0.72 (128) | 20, 11 | 0.105 | 0.097 | 1.09 | 1 |
| 1iarB | 0.76 | 23, 10 | 0.117 | 0.096 | 1.21 | 1 |
| 1ibrA | 0.97 (1215) | 45, 22 | 0.082 | 0.064 | 1.27 | 2 |
| 1ibrB | 0.82 | 53, 26 | 0.060 | 0.057 | 1.06 | 3 |
| 1icfA | 0.78 (4686) | 31, 17 | 0.120 | 0.095 | 1.26 | 1 |
| 1icfI | 1.18 | 23, 8 | 0.113 | 0.100 | 1.13 | 2 |
| 1igcA | 0.34 (1660) | 13, 5 | 0.136 | 0.107 | 1.27 | 1 |
| 1igcH | 1.13 | 15, 5 | 0.154 | 0.103 | 1.49 | 1 |
| 1iilA | 0.84 (3582) | 40, 19 | 0.079 | 0.075 | 1.06 | 2 |
| 1iilE | 0.98 | 40, 18 | 0.079 | 0.075 | 1.06 | 3 |
| 1ijeA | 0.02 (1495) | 53, 43 | 0.066 | 0.064 | 1.03 | 2 |
| 1ijeB | 0.41 | 47, 28 | 0.073 | 0.075 | 0.98 | 3 |
| 1iodA | 1.69 (930) | 22, 12 | 0.080 | 0.080 | 1.00 | 3 |
| 1iodG | 0.59 | 18, 8 | 0.102 | 0.093 | 1.10 | 1 |
| 1j2jA | 0.86 (1108) | 18, 11 | 0.124 | 0.099 | 1.25 | 1 |
| 1j2jB | 0.16 | 14, 10 | 0.113 | 0.102 | 1.10 | 1 |
| 1j34A | 1.71 (938) | 31, 17 | 0.077 | 0.074 | 1.04 | 4 |
| 1j34C | 0.55 | 22, 11 | 0.096 | 0.084 | 1.15 | 1 |
| 1jbuH | 1.35 (2861) | 32, 10 | 0.103 | 0.088 | 1.17 | 1 |
| 1jbuX | 0.00 | 14, 0 | - | 0.104 | - | - |
| 1jdhA | 0.22 (298) | 57, 39 | 0.060 | 0.057 | 1.06 | 3 |
| 1jdhB | 0.41 | 31, 19 | 0.065 | 0.064 | 1.02 | 3 |
| 1jdpA | 0.86 (263) | 51, 29 | 0.088 | 0.077 | 1.14 | 1 |
| 1jdpH | 0.16 | 18, 13 | 0.111 | 0.106 | 1.05 | 1 |
| 1jiwI | 0.87 (180) | 24, 11 | 0.096 | 0.091 | 1.05 | 2 |
| 1jiwP | 0.90 | 37, 16 | 0.103 | 0.085 | 1.22 | 2 |
| 1jpsH | 1.38 (1907) | 29, 11 | 0.082 | 0.087 | 0.93 | 3 |
| 1jpsT | 0.93 | 32, 15 | 0.078 | 0.083 | 0.94 | 3 |
| 1jtgA | 0.54 (3404) | 39, 22 | 0.097 | 0.082 | 1.18 | 1 |
| 1jtgB | 0.76 | 36, 21 | 0.090 | 0.079 | 1.14 | 2 |
| 1jthA | 0.26 (973) | 31, 20 | 0.063 | 0.060 | 1.04 | 3 |
| 1jthB | 0.60 | 28, 15 | 0.066 | 0.059 | 1.11 | 3 |
| 1jw9B | 0.79 (2028) | 36, 18 | 0.101 | 0.085 | 1.19 | 1 |
| 1jw9D | 0.67 | 26, 16 | 0.103 | 0.094 | 1.09 | 1 |
| 1jyoA | 0.02 (17) | 82, 35 | 0.075 | 0.053 | 1.41 | 2 |
| 1jyoF | 0.42 | 58, 18 | 0.058 | 0.052 | 1.11 | 5 |
| 1jzdA | 0.60 (607) | 31, 16 | 0.084 | 0.072 | 1.16 | 2 |
| 1jzdC | 0.80 | 35, 18 | 0.103 | 0.077 | 1.33 | 2 |
| 1k9oE | 0.94 (1126) | 35, 15 | 0.132 | 0.095 | 1.39 | 1 |
| 1k9oI | 1.13 | 19, 10 | 0.118 | 0.104 | 1.14 | 1 |
| 1kb5A | 1.32 (1870) | 34, 13 | 0.072 | 0.076 | 0.94 | 3 |
| 1kb5L | 1.35 | 33, 12 | 0.071 | 0.080 | 0.89 | 3 |
| 1ki1A | 0.42 (882) | 32, 20 | 0.100 | 0.082 | 1.22 | 1 |
| 1ki1B | 0.67 | 41, 18 | 0.080 | 0.072 | 1.11 | 3 |
| 1kshA | 0.89 (1110) | 24, 12 | 0.118 | 0.088 | 1.35 | 2 |
| 1kshB | 0.44 | 27, 17 | 0.088 | 0.084 | 1.05 | 2 |
| 1ktzA | 0.59 (439) | 10, 5 | 0.149 | 0.112 | 1.33 | 1 |
| 1ktzB | 0.36 | 16, 7 | 0.114 | 0.112 | 1.02 | 1 |
| 1kxvB | 0.86 (2938) | 30, 16 | 0.086 | 0.083 | 1.04 | 2 |
| 1kxvD | 1.18 | 26, 8 | 0.096 | 0.084 | 1.13 | 1 |
| 1kz7A | 0.86 (2655) | 50, 23 | 0.081 | 0.066 | 1.24 | 3 |
| 1kz7B | 0.69 | 35, 19 | 0.093 | 0.076 | 1.22 | 3 |
| 1l2iA | 0.50 (1700) | 15, 10 | 0.123 | 0.109 | 1.12 | 1 |
| 1l2iC | 0.00 | 9, 0 | - | 0.133 | - | - |
| 1l4dA | 1.28 (1537) | 24, 10 | 0.124 | 0.098 | 1.27 | 2 |
| 1l4dB | 0.28 | 24, 16 | 0.103 | 0.098 | 1.05 | 2 |
| 1l6xA | 0.94 (370) | 24, 13 | 0.118 | 0.104 | 1.14 | 1 |
| 1l6xB | 0.03 | 16, 14 | 0.102 | 0.102 | 1.00 | 1 |
| 1lfdA | 0.34 (1500) | 15, 9 | 0.118 | 0.102 | 1.16 | 1 |
| 1lfdB | 0.90 | 19, 9 | 0.095 | 0.097 | 0.98 | 2 |
| 1lk3A | 0.52 (3470) | 18, 8 | 0.110 | 0.103 | 1.07 | 1 |
| 1lk3H | 1.40 | 26, 10 | 0.096 | 0.098 | 0.98 | 1 |
| 1lpbA | 0.48 (281) | 20, 11 | 0.086 | 0.081 | 1.06 | 2 |
| 1lpbB | 1.24 | 25, 15 | 0.084 | 0.077 | 1.09 | 2 |
| 1lqvB | 0.70 (65) | 13, 8 | 0.130 | 0.116 | 1.12 | 1 |
| 1lqvD | 0.00 | 5, 0 | - | 0.153 | - | - |
| 1m4uA | 0.54 (899) | 9, 4 | 0.149 | 0.133 | 1.11 | 1 |
| 1m4uL | 1.10 | 11, 6 | 0.129 | 0.126 | 1.02 | 1 |
| 1m9eB | 0.40 (3000) | 20, 13 | 0.133 | 0.108 | 1.23 | 1 |
| 1m9eC | 0.07 | 13, 11 | 0.116 | 0.116 | 1.00 | 2 |
| 1mbxA | 0.69 (1204) | 27, 14 | 0.114 | 0.089 | 1.28 | 1 |
| 1mbxC | 0.89 | 21, 12 | 0.103 | 0.093 | 1.10 | 2 |
| 1mctA | 0.89 (1500) | 32, 16 | 0.131 | 0.097 | 1.35 | 1 |
| 1mctI | 0.31 | 15, 8 | 0.161 | 0.124 | 1.30 | 1 |
| 1mcvA | 1.09 (1492) | 38, 16 | 0.121 | 0.090 | 1.34 | 1 |
| 1mcvI | 0.40 | 20, 12 | 0.140 | 0.116 | 1.21 | 1 |
| 1melA | 1.42 (2778) | 21, 8 | 0.120 | 0.104 | 1.15 | 1 |
| 1melL | 0.95 | 25, 11 | 0.137 | 0.096 | 1.43 | 1 |
| 1mlcA | 1.38 (3218) | 23, 9 | 0.088 | 0.100 | 0.88 | 2 |
| 1mlcE | 1.15 | 20, 9 | 0.129 | 0.110 | 1.18 | 1 |
| 1mzwA | 0.97 (1500) | 18, 6 | 0.179 | 0.118 | 1.51 | 1 |
| 1mzwB | 0.24 | 12, 8 | 0.122 | 0.126 | 0.97 | 1 |
| 1ncaL | 1.39 (1799) | 31, 14 | 0.085 | 0.085 | 1.01 | 2 |
| 1ncaN | 0.45 | 28, 14 | 0.089 | 0.083 | 1.07 | 2 |
| 1nf3A | 0.65 (1885) | 36, 21 | 0.076 | 0.072 | 1.06 | 3 |
| 1nf3C | 1.18 | 28, 10 | 0.065 | 0.072 | 0.91 | 3 |
| 1nl0G | 0.68 (2069) | 8, 4 | 0.102 | 0.127 | 0.80 | 1 |
| 1nl0L | 1.48 | 23, 7 | 0.103 | 0.106 | 0.98 | 2 |
| 1nm1A | 0.08 (2508) | 29, 25 | 0.090 | 0.087 | 1.03 | 1 |
| 1nm1G | 0.75 | 28, 17 | 0.100 | 0.092 | 1.09 | 2 |
| 1nmbN | 0.56 (1658) | 19, 9 | 0.113 | 0.100 | 1.13 | 2 |
| 1nmbL | 1.37 | 21, 8 | 0.083 | 0.094 | 0.89 | 2 |
| 1nu9A | 1.35 (2809) | 57, 15 | 0.121 | 0.067 | 1.81 | 1 |
| 1nu9C | 0.34 | 58, 33 | 0.061 | 0.057 | 1.08 | 4 |
| 1nw9A | 1.06 (743) | 33, 13 | 0.097 | 0.087 | 1.12 | 2 |
| 1nw9B | 0.96 | 32, 11 | 0.093 | 0.079 | 1.17 | 2 |
| 1o6sA | 1.29 (952) | 58, 22 | 0.075 | 0.065 | 1.16 | 2 |
| 1o6sB | 1.02 | 35, 14 | 0.082 | 0.074 | 1.11 | 2 |
| 1oc0A | 1.21 (585) | 18, 6 | 0.116 | 0.096 | 1.21 | 1 |
| 1oc0B | 0.95 | 15, 5 | 0.131 | 0.114 | 1.15 | 1 |
| 1oebA | 0.85 (2144) | 18, 8 | 0.121 | 0.104 | 1.16 | 1 |
| 1oebD | - | 11, - | - | - | - | - |
| 1oeyA | 0.45 (208) | 18, 12 | 0.116 | 0.097 | 1.20 | 1 |
| 1oeyJ | 0.39 | 17, 8 | 0.134 | 0.100 | 1.34 | 1 |
| 1ofuA | 0.60 (1469) | 20, 9 | 0.107 | 0.101 | 1.06 | 1 |
| 1ofuX | 0.80 | 20, 9 | 0.107 | 0.092 | 1.16 | 1 |
| 1onqA | 0.62 (1672) | 46, 23 | 0.071 | 0.067 | 1.05 | 3 |
| 1onqB | 0.94 | 33, 14 | 0.077 | 0.072 | 1.07 | 2 |
| 1oo0A | 0.44 (1057) | 28, 18 | 0.088 | 0.083 | 1.06 | 2 |
| 1oo0B | 1.15 | 33, 15 | 0.081 | 0.080 | 1.02 | 3 |
| 1ophA | 1.34 (1251) | 13, 6 | 0.116 | 0.111 | 1.04 | 1 |
| 1ophB | 0.78 | 31, 17 | 0.135 | 0.101 | 1.33 | 1 |
| 1oryA | 0.88 (1265) | 51, 24 | 0.086 | 0.071 | 1.22 | 2 |
| 1oryB | 0.41 | 36, 19 | 0.073 | 0.074 | 0.99 | 3 |
| 1ospL | 1.33 (1625) | 21, 10 | 0.092 | 0.091 | 1.01 | 2 |
| 1ospO | 0.24 | 22, 13 | 0.096 | 0.093 | 1.04 | 2 |
| 1oy3B | 0.98 (2592) | 43, 19 | 0.066 | 0.055 | 1.21 | 3 |
| 1oy3D | 1.50 | 51, 20 | 0.067 | 0.061 | 1.10 | 3 |
| 1p5vA | 0.72 (566) | 52, 27 | 0.070 | 0.065 | 1.08 | 5 |
| 1p5vB | 0.01 | 52, 51 | 0.066 | 0.066 | 1.00 | 3 |
| 1pdkA | 1.03 (463) | 47, 21 | 0.088 | 0.069 | 1.28 | 2 |
| 1pdkB | 0.72 | 42, 21 | 0.082 | 0.074 | 1.11 | 2 |
| 1ppfE | 0.98 (1332) | 29, 13 | 0.135 | 0.094 | 1.43 | 1 |
| 1ppfI | 0.80 | 15, 6 | 0.140 | 0.103 | 1.35 | 1 |
| 1pxvA | 0.34 (44) | 36, 19 | 0.103 | 0.084 | 1.24 | 2 |
| 1pxvC | 0.07 | 30, 24 | 0.091 | 0.090 | 1.02 | 2 |
| 1q1sA | 0.00 (250) | 20, 0 | - | 0.076 | - | - |
| 1q1sC | 0.38 | 62, 42 | 0.067 | 0.060 | 1.10 | 2 |
| 1q40A | 0.25 (108) | 45, 27 | 0.073 | 0.076 | 0.96 | 3 |
| 1q40B | 0.42 | 48, 24 | 0.085 | 0.072 | 1.18 | 2 |
| 1qavA | 1.06 (1115) | 26, 10 | 0.120 | 0.093 | 1.29 | 1 |
| 1qavB | 1.31 | 21, 8 | 0.111 | 0.093 | 1.20 | 2 |
| 1qtxA | 0.52 (1500) | 52, 29 | 0.087 | 0.076 | 1.15 | 2 |
| 1qtxB | 0.06 | 19, 16 | 0.108 | 0.104 | 1.04 | 1 |
| 1r0rE | 0.74 (1445) | 31, 17 | 0.123 | 0.100 | 1.23 | 1 |
| 1r0rI | 0.77 | 15, 7 | 0.149 | 0.105 | 1.42 | 1 |
| 1r17A | 0.77 (126) | 32, 16 | 0.076 | 0.072 | 1.06 | 2 |
| 1r17B | 0.75 | 34, 16 | 0.073 | 0.071 | 1.04 | 2 |
| 1r3jA | 1.44 (1583) | 24, 9 | 0.092 | 0.099 | 0.93 | 2 |
| 1r3jC | 1.14 | 18, 9 | 0.117 | 0.114 | 1.02 | 1 |
| 1rewB | 1.20 (1810) | 23, 11 | 0.117 | 0.097 | 1.21 | 1 |
| 1rewD | 0.92 | 25, 13 | 0.093 | 0.089 | 1.05 | 2 |
| 1rj9A | 0.65 (2954) | 40, 19 | 0.078 | 0.070 | 1.12 | 3 |
| 1rj9B | 0.59 | 40, 20 | 0.077 | 0.067 | 1.16 | 3 |
| 1rjcA | 1.25 (2020) | 26, 10 | 0.088 | 0.098 | 0.90 | 2 |
| 1rjcB | 1.11 | 21, 9 | 0.136 | 0.095 | 1.43 | 1 |
| 1rkeA | 0.09 (104) | 37, 23 | 0.078 | 0.070 | 1.12 | 2 |
| 1rkeB | 0.46 | 31, 17 | 0.080 | 0.077 | 1.04 | 2 |
| 1rp3A | 0.95 (2982) | 58, 25 | 0.067 | 0.063 | 1.06 | 5 |
| 1rp3B | 0.00 | 43, 0 | - | 0.063 | - | - |
| 1s1qC | 0.61 (2877) | 18, 9 | 0.099 | 0.095 | 1.05 | 2 |
| 1s1qD | 0.52 | 17, 8 | 0.120 | 0.098 | 1.22 | 1 |
| 1s6cA | 0.62 (692) | 30, 16 | 0.102 | 0.084 | 1.22 | 1 |
| 1s6cB | 0.07 | 16, 12 | 0.102 | 0.108 | 0.95 | 2 |
| 1sbbA | 1.29 (1349) | 17, 8 | 0.128 | 0.113 | 1.13 | 2 |
| 1sbbB | 1.25 | 17, 6 | 0.098 | 0.101 | 0.97 | 2 |
| 1sbwA | 0.84 (1500) | 32, 17 | 0.122 | 0.097 | 1.26 | 1 |
| 1sbwI | 0.16 | 13, 8 | 0.131 | 0.117 | 1.12 | 1 |
| 1sg1A | 0.38 (1898) | 34, 20 | 0.068 | 0.063 | 1.08 | 2 |
| 1sg1X | 1.37 | 33, 14 | 0.066 | 0.063 | 1.04 | 3 |
| 1sgpE | 0.41 (313) | 26, 15 | 0.125 | 0.104 | 1.20 | 1 |
| 1sgpI | 0.76 | 14, 6 | 0.170 | 0.125 | 1.36 | 1 |
| 1shwA | 0.63 (288) | 16, 8 | 0.116 | 0.097 | 1.19 | 2 |
| 1shwB | 0.62 | 26, 12 | 0.118 | 0.094 | 1.27 | 1 |
| 1skoA | 0.38 (40) | 31, 19 | 0.092 | 0.084 | 1.10 | 3 |
| 1skoB | 0.37 | 30, 18 | 0.092 | 0.084 | 1.10 | 2 |
| 1slwA | 0.57 (3127) | 22, 13 | 0.108 | 0.097 | 1.11 | 3 |
| 1slwB | 0.89 | 32, 16 | 0.119 | 0.092 | 1.29 | 1 |
| 1sq2L | 0.92 (977) | 26, 12 | 0.122 | 0.097 | 1.26 | 1 |
| 1sq2N | 1.32 | 20, 11 | 0.113 | 0.105 | 1.07 | 1 |
| 1stfE | 0.77 (1493) | 29, 15 | 0.114 | 0.095 | 1.20 | 1 |
| 1stfI | 0.63 | 21, 11 | 0.124 | 0.105 | 1.18 | 2 |
| 1sv0B | 0.75 (347) | 15, 8 | 0.112 | 0.115 | 0.97 | 1 |
| 1sv0D | 0.63 | 16, 8 | 0.131 | 0.112 | 1.17 | 1 |
| 1svxA | 1.62 (881) | 21, 6 | 0.109 | 0.095 | 1.14 | 1 |
| 1svxB | 1.07 | 19, 7 | 0.127 | 0.109 | 1.16 | 2 |
| 1t0fA | 1.01 (103) | 39, 19 | 0.088 | 0.073 | 1.21 | 2 |
| 1t0fC | 0.17 | 31, 17 | 0.081 | 0.077 | 1.06 | 3 |
| 1t0jB | 0.06 (522) | 26, 21 | 0.098 | 0.098 | 1.00 | 2 |
| 1t0jC | 0.00 | 13, 0 | - | 0.118 | - | - |
| 1t0pA | 1.02 (229) | 21, 8 | 0.122 | 0.106 | 1.15 | 1 |
| 1t0pB | 0.80 | 16, 8 | 0.136 | 0.116 | 1.18 | 1 |
| 1t8oC | 0.78 (4748) | 34, 19 | 0.118 | 0.095 | 1.24 | 1 |
| 1t8oD | 1.01 | 15, 5 | 0.207 | 0.129 | 1.60 | 1 |
| 1ta3A | 1.34 (620) | 29, 10 | 0.081 | 0.082 | 0.98 | 2 |
| 1ta3B | 0.91 | 40, 19 | 0.106 | 0.077 | 1.37 | 2 |
| 1tabE | 0.93 (1456) | 34, 16 | 0.125 | 0.094 | 1.33 | 1 |
| 1tabI | 0.57 | 14, 7 | 0.142 | 0.113 | 1.26 | 1 |
| 1tawA | 0.72 (1412) | 30, 18 | 0.124 | 0.103 | 1.20 | 1 |
| 1tawB | 0.99 | 14, 5 | 0.210 | 0.137 | 1.54 | 1 |
| 1tgsI | 1.21 (1421) | 19, 6 | 0.135 | 0.102 | 1.32 | 1 |
| 1tgsZ | 0.96 | 33, 15 | 0.135 | 0.093 | 1.45 | 1 |
| 1th8A | 0.98 (431) | 24, 9 | 0.128 | 0.090 | 1.42 | 1 |
| 1th8B | 0.61 | 24, 15 | 0.116 | 0.094 | 1.24 | 1 |
| 1to2E | 0.75 (1452) | 32, 17 | 0.117 | 0.098 | 1.20 | 1 |
| 1to2I | 0.73 | 21, 13 | 0.123 | 0.107 | 1.15 | 1 |
| 1ttwA | 0.17 (15) | 29, 18 | 0.091 | 0.089 | 1.02 | 1 |
| 1ttwB | 0.00 | 14, 0 | - | 0.106 | - | - |
| 1tx4A | 0.89 (1643) | 32, 15 | 0.108 | 0.083 | 1.31 | 1 |
| 1tx4B | 0.48 | 29, 19 | 0.097 | 0.082 | 1.19 | 1 |
| 1ty4A | 0.13 (8) | 38, 31 | 0.079 | 0.076 | 1.04 | 2 |
| 1ty4C | 0.13 | 21, 17 | 0.095 | 0.088 | 1.08 | 2 |
| 1u0sA | 0.53 (1470) | 20, 11 | 0.111 | 0.103 | 1.08 | 1 |
| 1u0sY | 1.04 | 23, 8 | 0.130 | 0.102 | 1.28 | 1 |
| 1u6hA | 0.08 (44) | 43, 32 | 0.075 | 0.075 | 1.00 | 2 |
| 1u6hB | 0.06 | 22, 19 | 0.098 | 0.097 | 1.01 | 2 |
| 1u8tA | 1.05 (2553) | 17, 8 | 0.126 | 0.108 | 1.16 | 1 |
| 1u8tE | 0.00 | 9, 0 | - | 0.124 | - | - |
| 1uadA | 0.94 (1496) | 16, 9 | 0.117 | 0.104 | 1.12 | 1 |
| 1uadC | 0.87 | 19, 8 | 0.107 | 0.103 | 1.04 | 1 |
| 1ujzA | 0.86 (106) | 23, 10 | 0.116 | 0.099 | 1.18 | 1 |
| 1ujzB | 1.27 | 19, 6 | 0.138 | 0.102 | 1.36 | 1 |
| 1ukvG | 0.44 (1394) | 51, 29 | 0.072 | 0.061 | 1.17 | 3 |
| 1ukvY | 0.76 | 39, 21 | 0.087 | 0.067 | 1.29 | 3 |
| 1unlA | 1.04 (1502) | 40, 16 | 0.090 | 0.075 | 1.21 | 1 |
| 1unlD | 0.25 | 39, 31 | 0.085 | 0.076 | 1.11 | 2 |
| 1us7A | 0.60 (1371) | 21, 11 | 0.117 | 0.095 | 1.23 | 1 |
| 1us7B | 0.64 | 17, 10 | 0.102 | 0.098 | 1.04 | 2 |
| 1usuA | 1.05 (3278) | 18, 10 | 0.090 | 0.078 | 1.16 | 2 |
| 1usuB | 0.44 | 23, 13 | 0.085 | 0.080 | 1.06 | 2 |
| 1uxsA | 0.15 (1425) | 44, 35 | 0.064 | 0.066 | 0.96 | 3 |
| 1uxsB | 0.87 | 33, 15 | 0.079 | 0.075 | 1.05 | 2 |
| 1uzxA | 0.30 (1355) | 20, 14 | 0.105 | 0.102 | 1.02 | 1 |
| 1uzxB | 0.46 | 19, 9 | 0.107 | 0.098 | 1.09 | 1 |
| 1v18A | 0.21 (246) | 66, 38 | 0.062 | 0.057 | 1.08 | 4 |
| 1v18B | 0.06 | 30, 24 | 0.061 | 0.060 | 1.01 | 4 |
| 1v74A | 0.58 (13) | 26, 14 | 0.097 | 0.085 | 1.15 | 2 |
| 1v74B | 0.50 | 24, 6 | 0.135 | 0.085 | 1.59 | 1 |
| 1vf6A | 0.21 (56) | 24, 16 | 0.083 | 0.080 | 1.03 | 2 |
| 1vf6C | 0.22 | 28, 17 | 0.086 | 0.083 | 1.04 | 3 |
| 1vg0A | 0.40 (1953) | 45, 27 | 0.074 | 0.067 | 1.10 | 3 |
| 1vg0B | 0.61 | 34, 22 | 0.093 | 0.077 | 1.20 | 3 |
| 1vppV | 0.84 (327) | 18, 8 | 0.121 | 0.101 | 1.20 | 1 |
| 1vppX | 0.00 | 14, 0 | - | 0.115 | - | - |
| 1w98A | 0.91 (891) | 47, 24 | 0.071 | 0.067 | 1.06 | 2 |
| 1w98B | 0.91 | 52, 23 | 0.077 | 0.066 | 1.17 | 3 |
| 1wejF | 1.06 (1789) | 16, 8 | 0.104 | 0.098 | 1.06 | 1 |
| 1wejL | 1.35 | 23, 10 | 0.088 | 0.095 | 0.93 | 2 |
| 1wmhA | 0.21 (75) | 17, 13 | 0.112 | 0.104 | 1.08 | 1 |
| 1wmhB | 0.28 | 17, 10 | 0.112 | 0.103 | 1.09 | 1 |
| 1wmiA | 1.01 (34) | 56, 24 | 0.079 | 0.070 | 1.13 | 3 |
| 1wmiB | 0.00 | 44, 0 | - | 0.062 | - | - |
| 1wqjB | 0.54 (547) | 22, 14 | 0.102 | 0.089 | 1.14 | 2 |
| 1wqjI | 0.50 | 23, 11 | 0.102 | 0.098 | 1.04 | 1 |
| 1wwwW | 0.45 (1820) | 21, 13 | 0.085 | 0.085 | 1.00 | 2 |
| 1wwwX | 0.97 | 26, 11 | 0.100 | 0.088 | 1.14 | 2 |
| 1xb2A | 0.12 (1971) | 43, 31 | 0.073 | 0.064 | 1.14 | 3 |
| 1xb2B | 0.59 | 44, 27 | 0.067 | 0.058 | 1.15 | 3 |
| 1xd3A | 0.68 (1549) | 39, 21 | 0.093 | 0.087 | 1.07 | 2 |
| 1xd3B | 0.32 | 27, 15 | 0.096 | 0.094 | 1.03 | 1 |
| 1xg2A | 1.15 (507) | 31, 14 | 0.090 | 0.081 | 1.11 | 2 |
| 1xg2B | 1.27 | 35, 14 | 0.089 | 0.080 | 1.11 | 1 |
| 1xl3A | 0.22 (144) | 19, 13 | 0.094 | 0.090 | 1.04 | 2 |
| 1xl3C | 0.38 | 27, 14 | 0.091 | 0.091 | 1.00 | 1 |
| 1xt9A | 0.57 (1401) | 50, 24 | 0.086 | 0.075 | 1.14 | 2 |
| 1xt9B | 0.38 | 33, 20 | 0.088 | 0.083 | 1.06 | 2 |
| 1xx9A | 0.92 (4319) | 39, 18 | 0.127 | 0.090 | 1.42 | 1 |
| 1xx9C | 0.78 | 24, 13 | 0.120 | 0.097 | 1.23 | 2 |
| 1ycsA | 0.33 (483) | 20, 12 | 0.100 | 0.087 | 1.15 | 1 |
| 1ycsB | 1.21 | 23, 13 | 0.115 | 0.088 | 1.30 | 1 |
| 1ydrE | 0.86 (1500) | 36, 18 | 0.105 | 0.083 | 1.25 | 1 |
| 1ydrI | 0.02 | 18, 17 | 0.094 | 0.096 | 0.98 | 2 |
| 1yqvL | 1.35 (1660) | 25, 11 | 0.093 | 0.094 | 0.98 | 1 |
| 1yqvY | 1.18 | 21, 11 | 0.106 | 0.102 | 1.04 | 1 |
| 1yroA | 1.27 (936) | 17, 9 | 0.087 | 0.090 | 0.97 | 2 |
| 1yroB | 1.00 | 15, 7 | 0.115 | 0.093 | 1.24 | 1 |
| 1z7kA | 0.95 (1359) | 34, 16 | 0.121 | 0.091 | 1.33 | 1 |
| 1z7kB | 1.05 | 20, 6 | 0.128 | 0.101 | 1.26 | 1 |
| 2bf8A | 1.21 (1203) | 5, 2 | 0.164 | 0.142 | 1.16 | 1 |
| 2bf8B | 0.75 | 9, 4 | 0.197 | 0.154 | 1.28 | 1 |
| 2bo9A | 0.81 (622) | 37, 17 | 0.086 | 0.076 | 1.13 | 1 |
| 2bo9B | 0.61 | 33, 16 | 0.091 | 0.078 | 1.16 | 2 |
| 2kaiA | 0.79 (2113) | 28, 16 | 0.127 | 0.102 | 1.25 | 1 |
| 2kaiI | 0.99 | 14, 5 | 0.206 | 0.132 | 1.56 | 1 |
| 2ngrA | 0.43 (960) | 25, 16 | 0.105 | 0.085 | 1.23 | 1 |
| 2ngrB | 0.98 | 35, 15 | 0.099 | 0.079 | 1.24 | 2 |
| 2pccA | 1.03 (1339) | 15, 7 | 0.091 | 0.092 | 0.99 | 1 |
| 2pccB | 1.05 | 18, 6 | 0.098 | 0.092 | 1.06 | 1 |
| 2prgB | 0.47 (646) | 22, 13 | 0.103 | 0.094 | 1.10 | 2 |
| 2prgC | 0.07 | 14, 11 | 0.104 | 0.098 | 1.07 | 2 |
| 2ptcE | 0.80 (1385) | 33, 18 | 0.126 | 0.095 | 1.32 | 1 |
| 2ptcI | 0.98 | 15, 6 | 0.173 | 0.133 | 1.30 | 1 |
| 2sicE | 0.83 (1447) | 33, 17 | 0.120 | 0.096 | 1.26 | 1 |
| 2sicI | 1.01 | 15, 7 | 0.136 | 0.119 | 1.14 | 1 |
| 2tecE | 0.82 (1397) | 31, 17 | 0.120 | 0.099 | 1.21 | 1 |
| 2tecI | 0.63 | 18, 11 | 0.125 | 0.105 | 1.19 | 1 |
| 2trcB | 0.66 (371) | 65, 34 | 0.069 | 0.063 | 1.09 | 4 |
| 2trcP | 0.54 | 56, 27 | 0.070 | 0.059 | 1.19 | 3 |
| 3fapA | 1.16 (1483) | 17, 6 | 0.089 | 0.092 | 0.97 | 1 |
| 3fapB | 0.90 | 12, 5 | 0.103 | 0.095 | 1.08 | 1 |
| 3tpiI | 0.98 (1385) | 15, 6 | 0.174 | 0.133 | 1.31 | 1 |
| 3tpiZ | 0.80 | 49, 27 | 0.106 | 0.089 | 1.19 | 1 |
| 4htcH | 1.33 (1546) | 53, 14 | 0.125 | 0.071 | 1.76 | 2 |
| 4htcI | 0.30 | 33, 19 | 0.068 | 0.071 | 0.96 | 2 |

For the protein-protein complexes, the clustering is carried out separately on the interfacial regions of the two proteins forming the complex – the chain id of the component is mentioned after the PDB code.

For a few interfaces containing only a single or no conserved residues, the clustering could not be carried out and this is indicated with a dash (‘-‘) in the appropriate column. In case of 1oebD, multiple sequence alignment information for chain D is absent in 1oeb.hssp file and hence the clustering could not be performed for that interface.

a Average sequence entropy calculated over all interface residues. The number of homologous sequences in the HSSP database is indicated. For complexes, the number of aligned sequence is mentioned only once combining both the chains.

Table S2. Location of experimental hot spots within the conserved residue clusters in the interface

| PDB code & chain id | Protein complex^a^ | Statistics on clustering of conserved residues in the interface | | | Number of experimental ‘hot’ residues^b^ (number occurring within conserved clusters) | | |
| --- | --- | --- | --- | --- | --- | --- | --- |
|  |  | M_s,cons_ | M_s,int_ | ρ | ≥ 1 | ≥ 1.5 | ≥ 2 |
| 1a22A | Growth hormone | 0.089 | 0.073 | 1.22 | 6 (4) | 5 (4) | 3 (2) |
| 1a22B | Growth hormone receptor | 0.100 | 0.077 | 1.30 | 9 (7) | 8 (6) | 4 (3) |
| 1a4yA | Ribonuclease inhibitor | 0.073 | 0.067 | 1.09 | 5 (2) | 3 (2) | 2 (2) |
| 1a4yB | Angiogenin | 0.087 | 0.075 | 1.16 | 1 (0) | 1 (0) | 1 (0) |
| 1ahwAB | Ig Fab 5G9 | 0.083 | 0.087 | 0.95 | - | - | - |
| 1ahwC | Tissue factor | 0.079 | 0.085 | 0.93 | 5 (3) | 1 (0) | 1 (0) |
| 1aieA | p53 tetramer | 0.085 | 0.084 | 1.01 | 4 (4) | 4 (4) | 1 (1) |
| 1bp3A | Growth hormone | 0.081 | 0.072 | 1.13 | 7 (4) | 4 (4) | 2 (2) |
| 1bp3B | Prolactin receptor | 0.097 | 0.083 | 1.16 | - | - | - |
| 1brsA | Barnase | 0.112 | 0.096 | 1.16 | 6 (5) | 6 (5) | 6 (5) |
| 1brsD | Barstar | 0.121 | 0.107 | 1.12 | 5 (5) | 4 (4) | 3 (3) |
| 1bxiA | Immunity protein Im9 | 0.121 | 0.101 | 1.20 | 9 (5) | 8 (5) | 7 (4) |
| 1bxiB | Colicin E9 | 0.120 | 0.104 | 1.16 | - | - | - |
| 1cbwBC | Bovine chymotrypsin | 0.118 | 0.096 | 1.22 | - | - | - |
| 1cbwD | BPTI | 0.203 | 0.130 | 1.56 | 1 (0) | 1 (0) | 1 (0) |
| 1danLH | Factor VIIA | 0.064 | 0.056 | 1.15 | - | - | - |
| 1danTU | Tissue factor | 0.062 | 0.061 | 1.01 | 4 (4) | 3 (3) | 2 (2) |
| 1dkgAB | Nucleotide exchange factor (GRPE) | 0.070 | 0.065 | 1.07 | 9 (5) | 3 (2) | 1 (1) |
| 1dkgD | DNAK | 0.061 | 0.063 | 0.98 | - | - | - |
| 1dn2A | IgG1 Fc fragment | 0.122 | 0.102 | 1.20 | 3 (0) | 3 (0) | - |
| 1dn2E | Peptide | - | 0.128 | - | 2 (-) | 2 (-) | 2 (-) |
| 1dvfAB | Fv D1.3 | 0.094 | 0.091 | 1.03 | 13 (5) | 11 (4) | 6 (2) |
| 1dvfCD | Fv E5.2 | 0.086 | 0.091 | 0.94 | 9 (4) | 8 (3) | 3 (1) |
| 1emvA | Immunity protein Im9 | 0.122 | 0.101 | 1.21 | 11 (6) | 8 (5) | 7 (4) |
| 1emvB | Colicin E9 | 0.117 | 0.103 | 1.14 | - | - | - |
| 1fccA | IgG1 MO61 Fc | 0.106 | 0.104 | 1.01 | - | - | - |
| 1fccC | Protein G | 0.111 | 0.106 | 1.04 | 5 (4) | 4 (4) | 4 (4) |
| 1gc1C | CD4 | 0.090 | 0.088 | 1.02 | 3 (2) | - | - |
| 1gc1LH | Antibody | 0.117 | 0.107 | 1.09 | - | - | - |
| 1iarA | Interleukin-4 | 0.110 | 0.097 | 1.13 | 3 (3) | 2 (2) | 1 (1) |
| 1iarB | IL-4 receptor | 0.117 | 0.096 | 1.21 | - | - | - |
| 1jckA | T-cell antigen receptor | 0.065 | 0.062 | 1.05 | - | - | - |
| 1jckB | Staphylococcal enterotoxin C3 | 0.075 | 0.069 | 1.09 | 8 (3) | 6 (2) | 4 (2) |
| 1jdhA | Beta-catenin | 0.060 | 0.057 | 1.06 | - | - | - |
| 1jdhB | HTCF-4 | 0.065 | 0.064 | 1.02 | 1 (1) | 1 (1) | - |
| 1jrhI | IFNγ receptor | 0.106 | 0.104 | 1.02 | 6 (2) | 6 (2) | 5 (2) |
| 1jrhLH | Antibody A6 | 0.087 | 0.093 | 0.94 | 11 (1) | 7 (0) | 3 (0) |
| 1jtgA | β-lactamase | 0.094 | 0.082 | 1.14 | 1 (1) | - | - |
| 1jtgB | β-lactamase inhibitory protein | 0.090 | 0.079 | 1.14 | 1 (1) | 1 (1) | - |
| 1nmbN | N9 Neuraminidase | 0.113 | 0.099 | 1.14 | - | - | - |
| 1nmbLH | Fab NC10 | 0.082 | 0.091 | 0.90 | 1 (0) | 1 (0) | - |
| 1p2jA | Trypsinogen | 0.128 | 0.103 | 1.24 | - | - | - |
| 1p2jI | PTI | 0.175 | 0.132 | 1.33 | 4 (2) | 4 (2) | 4 (2) |
| 1vfbAB | IgG1-κ D1.3 Fv | 0.097 | 0.095 | 1.03 | 5 (1) | 3 (0) | 2 (0) |
| 1vfbC | HEL | 0.115 | 0.100 | 1.14 | 4 (1) | 2 (0) | 1 (0) |
| 2ptcE | β-trypsin | 0.126 | 0.095 | 1.32 | - | - | - |
| 2ptcI | Trypsin inhibitor | 0.173 | 0.133 | 1.30 | 1 (0) | 1 (0) | 1 (0) |
| 3hfmLH | HYHEL-10 IgG1 Fab | 0.081 | 0.090 | 0.90 | 11 (4) | 10 (4) | 9 (4) |
| 3hfmY | HEL | 0.115 | 0.092 | 1.25 | 6 (5) | 4 (4) | 3 (3) |
| 3hhrA | Human growth hormone | 0.070 | 0.061 | 1.13 | 8 (6) | 5 (4) | 3 (2) |
| 3hhrBC | hGH binding protein | 0.063 | 0.057 | 1.10 | 10 (6) | 8 (6) | 5 (3) |
| Overall extent of localization of experimental hot spot residues within conserved residue clusters^c^ | | | | | 196 (106) | 146 (83) | 95 (55) |

a The names of the protein components comprising each complex have been provided and the chain id of that component is given along with the PDB code in the first column.

b ‘Hot’ residues are defined in three ways with ΔΔG values ≥ 1, or ≥ 1.5, or ≥ 2 kcal/mol.

Components for which experimental alanine scanning data are not available are marked with ‘-‘.

c If antibody molecules are ignored when calculating the number of experimental ‘hot’ residues that are located within clusters of conserved residues, the statistics are 143 (91), 103 (72), and, 72 (48), for residues with ΔΔG values ≥ 1, ≥ 1.5, and, ≥ 2 kcal/mol, respectively.

Table S3. Distribution of 462 alanine scanned interface residues [39] among the seven residue classes [15] (indicating that the residues chosen for Ala-scanning experiments cover all the groups and are not biased towards one (or a few) groups). The division of the subset of hot spot residues (ΔΔG ≥ 1.5 kcal/mol) among the seven classes is also shown indicating the absence of domination by any single group.

| Amino acid class^a^ | Number (and percentage) in entire ΔΔG dataset | Number (and percentage) of hot spots (ΔΔG ≥ 1.5 kcal/mol) |
| --- | --- | --- |
| Val, Leu, Ile, Met, Cys | 63 (13.6) | 22 (14.2) |
| Gly, Ser, Thr | 73 (15.8) | 8 (5.2) |
| Asp, Glu | 76 (16.5) | 26 (16.8) |
| Asn, Gln | 54 (11.7) | 12 (7.7) |
| Arg, Lys | 84 (18.2) | 30 (19.4) |
| Pro, Phe, Tyr, Trp | 92 (19.9) | 52 (33.5) |
| His | 20 (4.3) | 5 (3.2) |

a Classification taken from Guharoy and Chakrabarti (2005) [15].

Figure S1. Representative examples of interfaces showing the clustered nature of evolutionarily conserved residues (conserved residues in orange, the rest of the interface residues in white). (A) Homodimer with PDB code 1dqs, chain A (ρ = 1.40), (B) protein-protein complex with PDB code 1azz, chain A (ρ = 1.40), and, (C) protein complex with PDB code 1v74, chain B (ρ = 1.59).

(A)


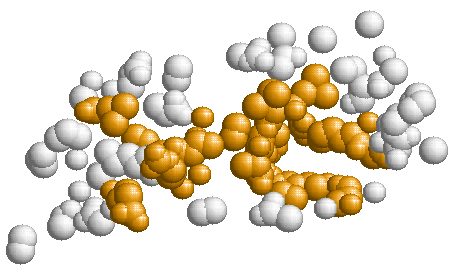


(B)


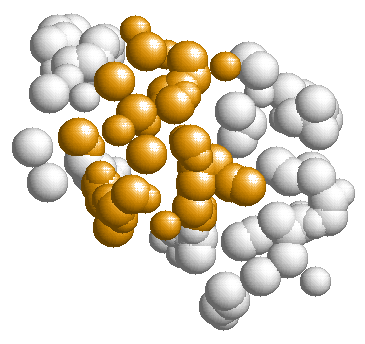


(C)


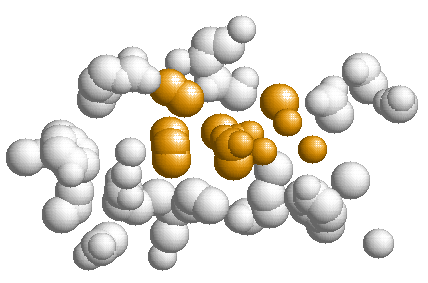


Figure S2. Plots of Ms,cons versus Ms,int for (A) homodimers, and (B) protein-protein complexes, respectively. This figure is similar to Figure 1, except that the subset of conserved residues in each interface is selected using a more stringent criteria (i.e., s < (<s>_int_ – σ), where, ‘s’ is the sequence entropy for an individual interface residue, <s>_int_ is the average sequence entropy calculated over all interface residues, and, σ, is the standard deviation of sequence entropies over all interface residues.

(A)

(B)

Figure S2 (contd.). Ms,cons versus Ms,int for (C) homodimers, and (D) protein-protein complexes, respectively, defining the conserved residues as those with s = 0.0 (i.e., the fully conserved ones).


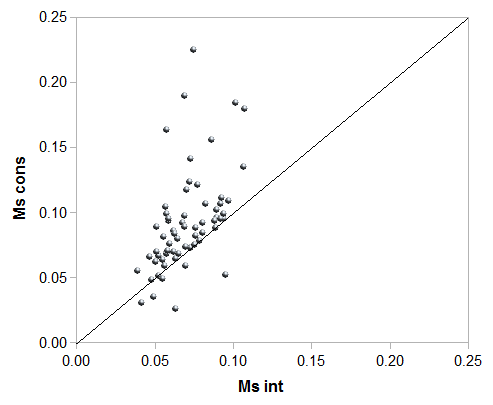
(C)

(D)


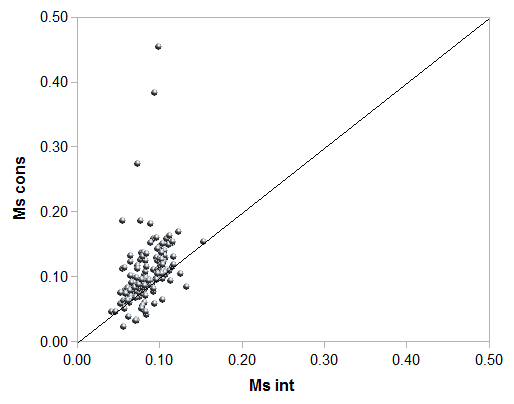


Figure S3. Plots of M_s,cons_ versus <M_s,random_> for all interfaces in (A) homodimers, and, (B) protein-protein complexes.

(A)

(B)

Figure S4. Number of interface residues and conserved residues as a function of interface area (per subunit) in (A) homodimers, and (B) protein-protein complexes. Correlation coefficients are, in (A) 0.98 for all interface residues and 0.89 for conserved residues; the corresponding values in (B) are 0.80 and 0.72.

(A)

(B)

Figure S5. Multiple clusters of evolutionary conserved residues in protein interfaces. (A) In the complex between Rac and RhoGDI (PDB code 1ds6), the interface of the latter contains two well-clustered regions of conserved residues. (B) In the complex between internalin A and E-cadherin (1o6s), the interface of the former has two conserved clusters. (C) Three conserved clusters in the interface of glucosamine 6-phosphate synthase (1mor). (D) Four clusters in the interface of human cellular coagulation factor XIII (1f13). Figures on the left show the protein domains as cartoons (green) and the interface region as spheres; those on the right have the same orientation but show only the interface region.


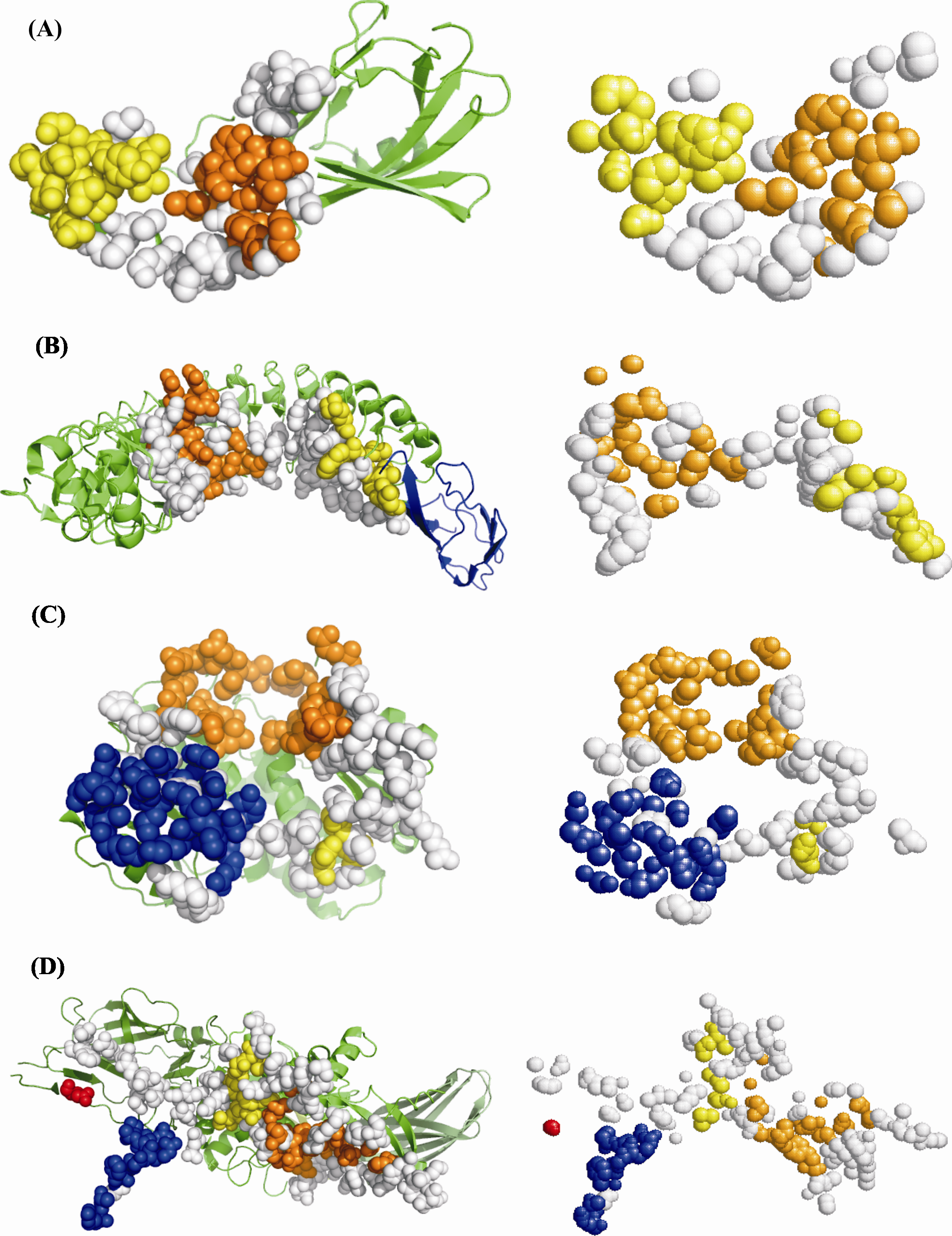


Figure S6. Distribution of cluster size (the number of interface residues in each conserved cluster) in (A) homodimers, and, (B) protein complexes.

(A)


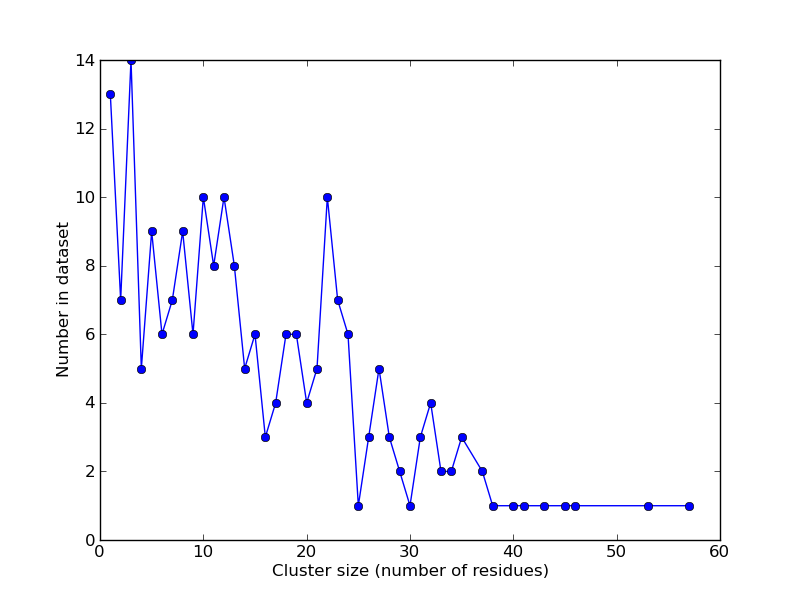


(B)


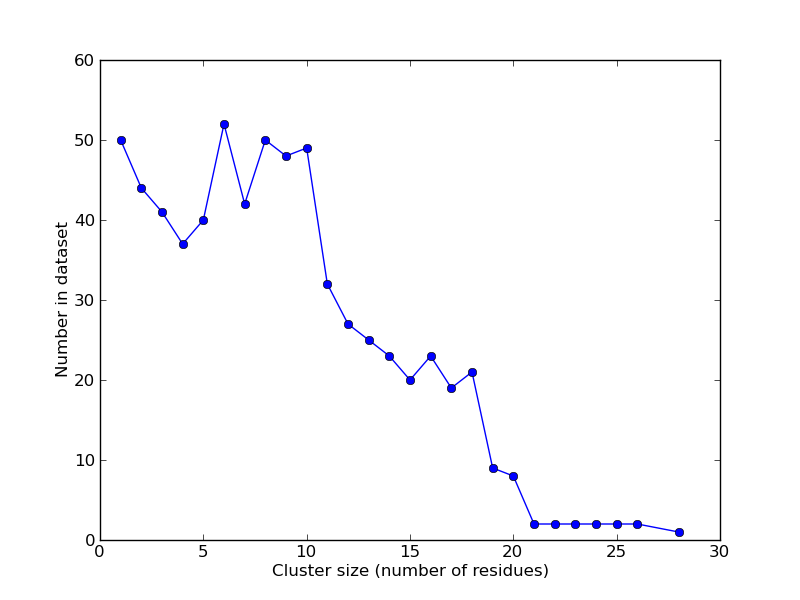


Figure S7.The level of sequence conservation of residues subjected to alanine scanning experiments. Plot of ΔΔG vs. sequence conservation (measured as sequence entropy; calculated using Eq. 1) showing that the ala-scanned residues cover a large range of entropy values (on the x-axis: from entropy=0.0, fully conserved, to poorly conserved, large entropy), and their distribution is pretty uniform. The degree of correlation is poor between ΔΔG values and sequence entropy.


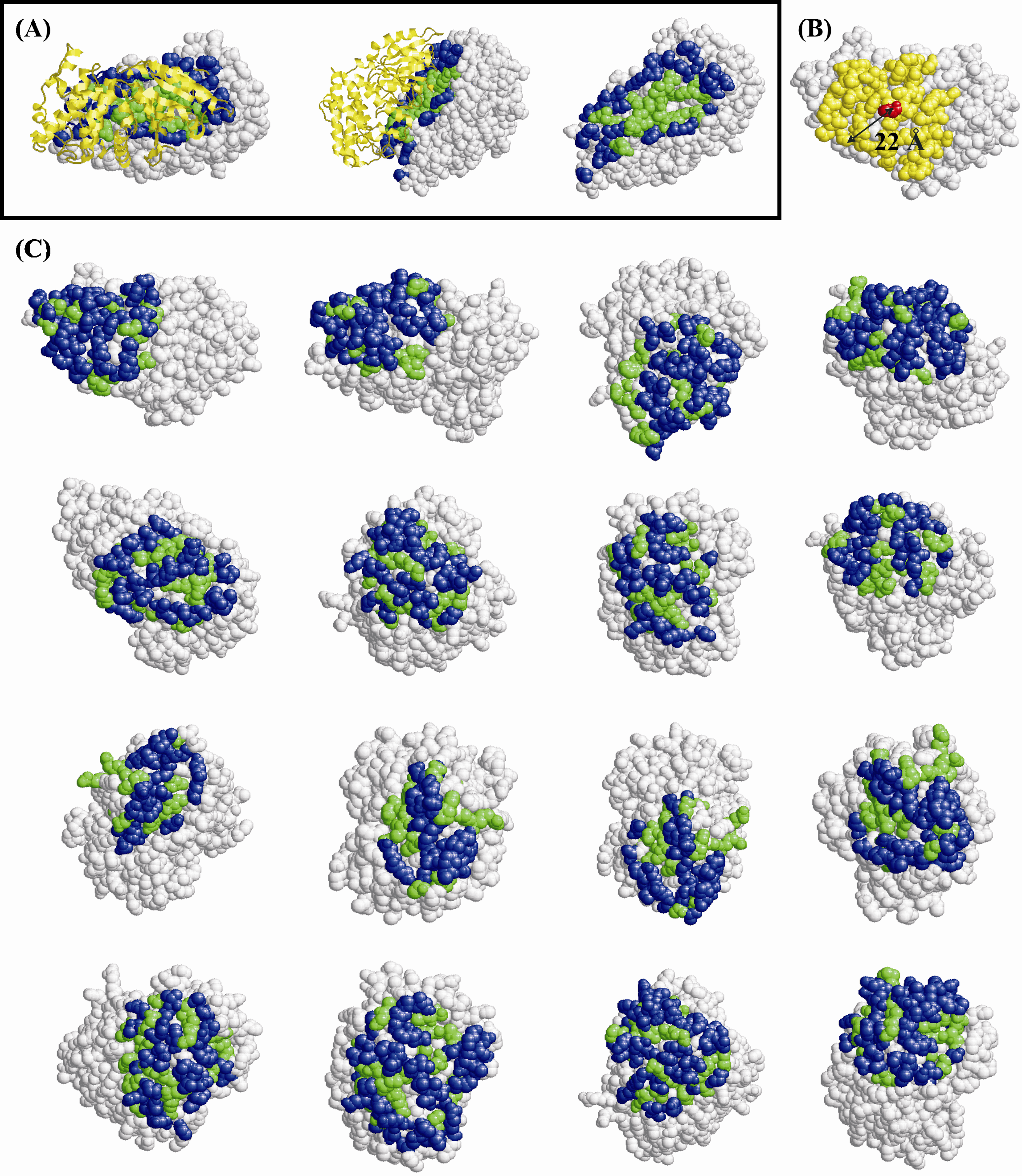
Figure S8. Comparison of the clustering of conserved residues within the subunit interface and other surface patches of the homodimeric enzyme 3-dehydroquinate synthase (1DQS). (A) One subunit of the molecule is shown in spacefill (grey), its partner in cartoon representation (yellow) in two different orientations. Conserved interface residues are colored green, the remaining interface residues are in blue. The second subunit (on top) is removed in the third view to clearly show the clustered nature of the conserved residues within the interface. (B) Diagram showing the construction of surface patches around each surface residue using a fixed cutoff of 22 Å (see text for details). (C) Sixteen different surface patches of the monomer (in grey spacefill) are shown; in each of them the conserved residues (green) are scattered over the entire patch.

Figure S9. Plot of Ms,cons versus Ms,int for interfaces from the bound forms 124 protein complexes described in the Protein-protein Docking Benchmark version 3.0 [38]. The two components of each complex are considered separately. Conserved residues are selected as those with individual sequence entropy less than the average value for the entire interface (same as in Figure 1); antibodies are marked as red triangles to show that the clustering of conserved interface residues does not hold true for a large majority of these interfaces (points below the diagonal).

Average values are: Interfaces excluding antibody, <Ms int> = 0.089±0.02, <Ms cons> = 0.1±0.02, <ρ> = 1.15±0.1 (P = 2.0E-10). For antibodies alone, <Ms int> = 0.092±0.01, <Ms cons> = 0.092±0.02, <ρ> = 1.0±0.1 (P=0.53). These are very similar to the values given in Table 1.


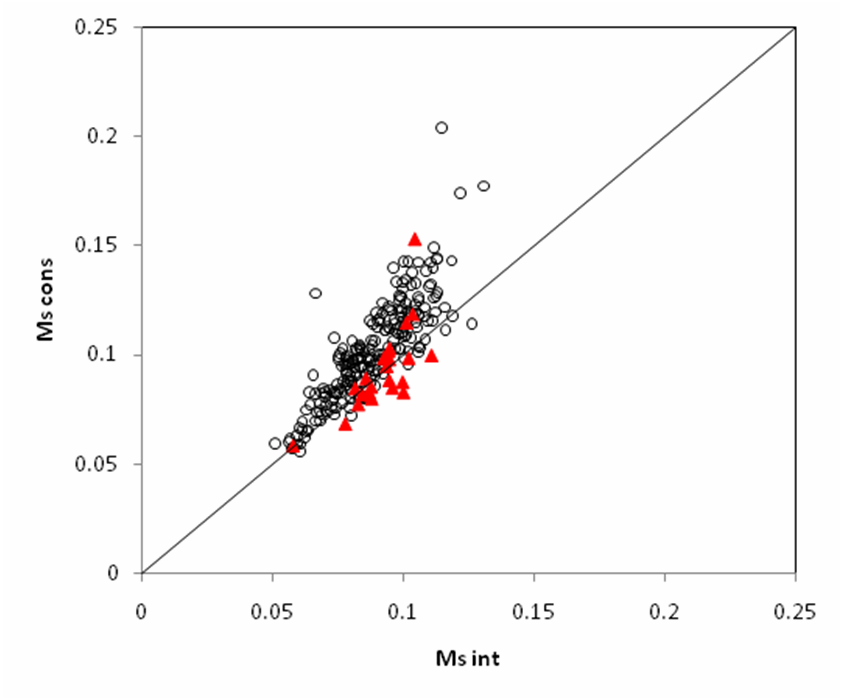

Supplement: Additional file 1 — The file contains three tables (numbered S1 to S3), and nine figures (numbered S1 to S9). Table S1. Values of the parameters indicating the clustering of conserved residues in individual interfaces. Table S2. Location of experimental hot spots within the conserved residue clusters in the interface. Table S3. Distribution of 462 alanine scanned interface residues among the seven residue classes. Figure S1. Representative examples of interfaces showing the clustered nature of evolutionarily conserved residues. Figure S2. Plots of Ms,cons versus Ms,int. Figure S3. Plots of Ms,cons versus < Ms,random>. Figure S4. Number of interface residues and conserved residues as a function of interface area. Figure S5. Multiple clusters of evolutionary conserved residues in protein interfaces. Figure S6. Distribution of cluster size. Figure S7. The level of sequence conservation of residues subjected to alanine scanning experiments. Figure S8. Comparison of the clustering of conserved residues within the subunit interface and other surface patches. Figure S9. Plot of Ms,cons versus Ms,int for interfaces from the bound forms 124 protein complexes described in the Protein-protein Docking Benchmark version 3.0. [file 1471-2105-11-286-S1.DOC]
